# An improved memory-based collaborative filtering method based on the TOPSIS technique

**DOI:** 10.1371/journal.pone.0204434

**Published:** 2018-10-04

**Authors:** Hael Al-bashiri, Mansoor Abdullateef Abdulgabber, Awanis Romli, Hasan Kahtan

**Affiliations:** Faculty of Computer Systems & Software Engineering, Universiti Malaysia Pahang, Kuantan, Pahang, Malaysia; Education University of Hong Kong, CHINA

## Abstract

This paper describes an approach for improving the accuracy of memory-based collaborative filtering, based on the technique for order of preference by similarity to ideal solution (TOPSIS) method. Recommender systems are used to filter the huge amount of data available online based on user-defined preferences. Collaborative filtering (CF) is a commonly used recommendation approach that generates recommendations based on correlations among user preferences. Although several enhancements have increased the accuracy of memory-based CF through the development of improved similarity measures for finding successful neighbors, there has been less investigation into prediction score methods, in which rating/preference scores are assigned to items that have not yet been selected by a user. A TOPSIS solution for evaluating multiple alternatives based on more than one criterion is proposed as an alternative to prediction score methods for evaluating and ranking items based on the results from similar users. The recommendation accuracy of the proposed TOPSIS technique is evaluated by applying it to various common CF baseline methods, which are then used to analyze the MovieLens 100K and 1M benchmark datasets. The results show that CF based on the TOPSIS method is more accurate than baseline CF methods across a number of common evaluation metrics.

## Introduction

Traditional information outlets—including friends, newspapers, advertisements, and mass media—have been increasingly supplanted by the Internet as a source for advice and guidance in decision making. Although the Internet is a powerful resource, the vast quantity of data available online can make it difficult to obtain the information needed to make decisions efficiently [1]. Research on the problem of online information overload has led to the development of tools, such as recommendation systems (RSs), that assist users in effective decision making [2]. RSs make suggestions to users based on preferences inferred from prior selections [3–10], thus reducing the time and effort required to make online selections [11]. This process depends on users’ historical behavior and involves the construction of user profiles for comparison with those of other users to locate his/her nearest neighbors in terms of preference. This process results in a list of items that are predicted to be most preferred by the user [3, 6, 7, 12].

Moreover, RS studies are of significant interest to a variety of entities and have been the focus of intensive academic and commercial research [13, 14]. Many commercial and nonprofit websites, including Amazon, eBay, and Lazada, use RS to assist their customers in purchasing items by making suggestions based on their previous selections and those of the most-similar customers. Such recommendations have become an integral aspect of e-commerce platforms and are used to personalize the shopping experience [15].

In general, RSs can be classified as either content-based, collaborative filtering (CF)-based, or a hybrid of the two [6, 8, 12, 14, 16–21]. In content-based approaches, items are recommended to the target user by comparing the information content of his/her past item selections with the content of items in the database [6, 22–26]. In contrast, CF-based systems propose items based on an analysis of user feedback along with the preferences of similar users [3, 27–32]; this additional robustness makes CF the most widely used and successful RS method. CF approaches can be further classified into model- and memory-based techniques [1, 33–35]. Model-based approaches apply a pre-built model for predicting user preferences, whereas memory-based approaches (also known as neighbor-based models) access entire databases of user-provided ratings to find correlations between users/items. Memory-based recommendation algorithms can generally be further subdivided into user- and item-based approaches [1, 12, 32, 36].

This paper addresses the application of user-based algorithms. Such algorithms use two key processes—similarity computing and prediction. In the computing process, the system seeks to find relationships between users, and those who are strongly correlated are designated as the neighbors of the target user. Any items rated by these neighbors that have not yet been purchased or obtained by the target user are then assembled into a set of candidate items. In the second process, the system predicts a user score for each item in the candidate set and promotes the highest-rated items as recommendations. This process of evaluating and ranking candidate items is therefore quite significant to the performance accuracy of the CF algorithm. Thus, the essential problem in information filtering is calculating whether a specific item is likely to be of interest to a user. The outcome of this process can be either Boolean (yes or no) or a score representing the degree to which the item is of interest. Unfortunately, most studies on improving the accuracy of conventional CF systems have focused solely on enhancing the similarity measure [3, 5, 6, 11, 37–51]. In contrast, improving the prediction algorithm has been somewhat neglected, even though it is of similar importance in improving memory-based CF recommendations [33, 52]. Prediction algorithms produce user preference scores for items using common aggregation methods. In this paper, we propose a method for enhancing the accuracy of memory-based CF recommendations by replacing the conventional prediction algorithm with TOPSIS, which is one of the most frequently used techniques for the evaluation and ranking of multiple alternatives.

As mentioned above, the majority of studies on enhancing the accuracy of CF have focused on improving the similarity measure, with relatively few investigating the prediction score models, even though these are of similar importance [53]. In this study, we investigated the use of TOPSIS as an alternative to prediction models for improving the accuracy of user-based CF. The proposed method applies TOPSIS in the evaluation and sorting of items rated by nearest-neighbor users to produce a set of Top-M ranked recommendations. The TOPSIS method can be described as a measurement technique based on the use of defined criteria to rank sets of alternatives, and is widely used as a tool in decision support problems. TOPSIS is useful in evaluating, sorting, and selecting from a variety of available options [54].

The remainder of this paper is organized as follows. The following section provides an overview of traditional collaborative RSs, discusses relevant memory-based CF methods, and outlines the TOPSIS method. The proposed TOPSIS-based recommendation method is then presented, before the experimental methodology and results are discussed. Finally, the conclusions to this study are provided with suggestions for future work.

## Related work

### Collaborative filtering techniques

The term “collaborative filtering” was first applied by Goldberg to the Tapestry recommender system [55], and CF has since become one of the most widely used techniques for providing service recommendations to users online [11, 56, 57]. As discussed in the Introduction, CF can be either model-based or memory-based.

In model-based approaches, a pre-built model is used to predict user preferences [14]. The most widely used approaches, involving the use of Bayesian networks or cluster models, were proposed in [58, 59]; the use of latent factor models was subsequently proposed in [60, 61]. Whereas Memory-based approaches compute the correlations between users and items to produce a preference score that predicts the likelihood of a user acquiring an item in the future and provide corresponding recommendations. User- and item-based algorithms are the most common types of memory-based recommendation methods [1, 12, 36]. User-based methods generate recommendations according to the similarities between users [29], whereas item-based methods compute similarities within a space of items to find strong relationships with items that have already been rated by an active user [29, 62].

User-based CF, the first automated CF method to be developed [28], was initially applied in the Group Lens Usenet article recommender [3], and is currently used in the BellCor video and Ringo music recommenders [4, 5]. This technique essentially involves four stages:

- user-to-user correlations are applied to find the most similar users to a target user (the neighbors) [29];
- after collecting items rated by neighbors, those that have already been obtained by the target user are removed, leaving a set of candidate items;
- a degree of preference score is generated to determine the likelihood of future purchase by the target user for each candidate item;
- based on their respective prediction scores, the items are ranked and a list of recommendations comprising the items with the highest ranks is generated.

In item-based CF, which was first proposed by Karypis and Sarwar [29], similarities among items are calculated according to other users’ evaluations. Generally, item-based CF follows the same steps as the user-based method, except that relationships are calculated across the space of items.

Common similarity measures and their limitations were discussed in [63, 64]. In the next section, we briefly present the most commonly used conventional memory-based CF methods.

### Baseline memory-based CF methods

i Pearson’s Correlation Coefficient

Resnick and Iacovou [3] used Pearson’s correlation coefficient (PCC) to find correlations among users in an approach that has become popular in memory-based CF. However, the PCC method can be inaccurate when the data are sparse, as missed ratings make it difficult to find correlations between users. This leads to high/low similarities and, therefore, weak recommendations [11, 65, 66]. The relationship among users can be defined as:

$${S(x,y)}^{PCC}=\frac{\sum_{i\in I_{xy}}\left(r_{x,i}-\bar{r_{x}})(r_{y,i}-\bar{r_{y}}\right)}{\sqrt{\sum_{i\in I_{xy}}{\left(r_{x,i-}\bar{r_{x}}\right)}^{2}}\sqrt{\sum_{i\in I_{xy}}{{(r}_{y,i}-\bar{r_{y}})}^{2}}},$$ (1)

where *S*(*x*,*y*)^*PCC*^ is the similarity between users x and y, *I*_*xy*_ represents the set of items that are rated by both x and y, $\bar{r}x$ and $\bar{r}y$ denote the average ratings by users x and y, respectively, and *r*_*x*,*i*_ denotes the rating value given to item *i* by user *x*.

ii Constrained Pearson Correlation

The RINGO recommender was developed to provide users with recommendations of music albums and artists. Under RINGO, users provide feedback on a nominal scale from one (“strong dislike”) to seven (“strong like”), with a neutral value (“neither like nor dislike”) in the middle of the scale. Based on the increasing number of RINGO users, Shraddhanand and Mae [5] proposed the constrained Pearson correlation (CPCC) approach to replace the average rating variables used by PCC approaches with the median value of a scale of positive and negative ratings. The correlation is calculated as:

$${S(x,y)}^{CPCC}=\frac{\sum_{i\in I_{xy}}\left(r_{x,i}-r_{m})(r_{y,i}-r_{m}\right)}{\sqrt{\sum_{i\in I_{xy}}{\left(r_{x,i-}r_{m}\right)}^{2}}\sqrt{\sum_{i\in I_{xy}}{{(r}_{y,i}-r_{m})}^{2}}},$$ (2)

where *r*_*m*_ denotes the median value of the rating scale.

iii Cosine method

The cosine method is a vector-space model that applies a linear algebra approach to define the relationships between pairs of users [6] as vectors, with user similarities computed as the cosine distance between each pair of rating vectors. This correlation is defined as:

$${S(x,y)}^{Cosine}=\frac{\sum_{i\in I_{xy}}\left(r_{x,i})(r_{y,i}\right)}{\sqrt{\sum_{i\in I_{xy}}{\left(r_{x,i}\right)}^{2}}\sqrt{\sum_{i\in I_{xy}}{{(r}_{y,i})}^{2}}},$$ (3)

iv Jaccard method

Koutrika and Bercovitz [58] proposed the Jaccard method to compute the correlations between pairs of users. The Jaccard method only considers the number of co-ratings for each user pair to define their relationship. Two users will have a strong correlation if they have similar rating patterns, and vice versa. However, the Jaccard computation process does not consider the absolute values of ratings [44, 45]. Formally, the similarity between users *x* and *y* is given by:

$${S(x,y)}^{Jaccard}=\frac{\left|I_{xy}\right|}{\left|I_{x}\cup I_{y}\right|},$$ (4)

where |*I*_*x*_ ∪ *I*_*y*_| represents the union set of items rated by users x and y.

v Sigmoid function-based PCC

Jamali and Ester [46] used a sigmoid function to decrement the similarity values between items for which few users have rated both items. The sigmoid function-based PCC (SPCC) approach produces similarity values in the range [0, 1] using the following formulation:

$${S(x,y)}^{SPCC}={s(x,y)}^{pcc}.\frac{1}{1+{exp}^{\left(-\frac{\left|I\right|}{2}\right)}},$$ (5)

However, a pair of users with similar ratings can still have a low similarity under this approach. For example, two users with ratings vectors of *u*1 = (4,3,5,4) and *u*2 = (4,3,3,4) will have very similar ratings but an SPCC similarity of zero.

vi Jaccard and mean squared difference measure

Bobadilla and Serradilla [39] hybridized the Jaccard [67] method with a mean squared difference approach [5] to produce the JMSD measure, which is computed as follows:

$${S(x,y)}^{JMSD}=s{\left(x,y\right)}^{MSD}+s{\left(x,y\right)}^{Jacc},$$ (6)

where

$${S(x,y)}^{MSD}=\frac{\left|I_{xy}\right|}{\sum_{i\in I}{\left(r_{x,i-}r_{y,i}\right)}^{2}}.$$

The JMSD approach addresses the respective drawbacks of the Jaccard and mean squared difference approaches, but suffers from the cold-start problem, does not consider the credibility of common ratings, and is vulnerable to local information and the utilization of rating problems [45].

vii New heuristic similarity measure (proximity–significance–singularity)

Liu and Hu [66] analyzed the drawbacks of Principles in Pattern approaches [38] and proposed an improved version called the new heuristic similarity measure (NHSM). The NHSM model considers three user rating factors—proximity, significance, and singularity (PSS)—and combines local context information on these ratings with the global preferences of user ratings to alleviate the cold-start problem [68]. However, NHSM only considers co-rated items in identifying relationships between users [44]. The measure is defined as:

$${S(x,y)}^{PSS}=\sum_{i\in I}PSS(r_{x,i},r_{y,i}),$$ (7)

where *PSS*(*r*_*x*,*i*_,*r*_*y*,*i*_) is the PSS value of users *x* and *y*, which is calculated as:

$$PSS(r_{x,i},r_{y,i})=Proximity(r_{x,i},r_{y,i})*Significance\;(r_{x,i},r_{y,i})*Singularity(r_{x,i},r_{y,i}).$$

The individual aspects are given by:

$$Proximity(r_{x,i},r_{y,i})=1-\frac{1}{1+\exp\left(-|r_{x,i}-r_{y,i}|\right)},$$ (8)

$$Significance(r_{x,i},r_{y,i})=\frac{1}{1+exp(-|r_{x,i}-r_{med}|*|r_{y,i}-r_{med}|)},$$ (9)

$$Singularity(r_{x,i},r_{y,i})=1-\frac{1}{1+\exp\left(-|\frac{r_{x,i}-r_{y,i}}{2}-\mu_{i}|\right)},$$ (10)

Liu and Hu further combined the PSS measures with the Jaccard measure to address the problem of small proportions of common ratings. This so-called JPSS measure is defined as:

$${S(x,y)}^{JPSS}={S(x,y)}^{Jacc}*{S(x,y)}^{PSS},$$ (11)

To account for cases arising when different rating preferences are provided by different users (i.e., high ratings provided by some and low ratings by others), they also developed a measure of user preference based on the rating mean and standard variance:

$${S(x,y)}^{URP}=1-\frac{1}{1+\exp\left(-|\mu_{x}-\mu_{y}|*|\sigma_{x}-\sigma_{y}|\right)},$$ (12)

where *σ*_*x*_ and *μ*_*x*_ are the mean rating and standard variance for user x, respectively, which are defined as:

$$\sigma_{x}=\frac{\sqrt{\sum_{i\in I_{x}}{{(r}_{x,i}-\bar{r_{x}})}^{2}}}{\left|I_{x}\right|},$$ (13)

$$\mu_{x}=\frac{\sqrt{\sum_{i\in I_{x}}{(r}_{x,i})}}{\left|I_{x}\right|},$$ (14)

Their final formalization combined the JPSS and user rating preference metrics into the improved new heuristic similarity model, or improved NHSM, which is defined as:

$${S(x,y)}^{NHSM}={S(x,y)}^{JPSS}*{S(x,y)}^{URP},$$ (15)

Note that prediction algorithms have not been mentioned in the above discussion, which instead has focused on improving the accuracy of memory-based CF through the development of similarity methods. In general, there are a number of mechanisms in the generation of recommendations that can predict the score for target user *x* with respect to item *i*. Many such methods involve aggregation (see Table 1) [69]. In this paper, we propose replacing such conventional methods with the TOPSIS approach to obtain improved recommendations.

**Table 1:** Aggregation methods.

Algorithm	Formula
Average method	$P_{x,i}=1/{|G}_{x,i}|\sum_{y\in G_{x,i}}r_{y,i}$, where *G*_*x*,*i*_ ≠ ∅
Weighted sum method	Px,i=∑y∈Gx,is(x,y)*ry,i∑y∈Gx,is(x,y), where *G*_*x*,*i*_ ≠ ∅
Adjusted weighted method (Deviation-From-Mean)	Px,i=rx¯+∑y∈Gx,is(x,y)*(ry,i−ry¯)∑y∈Gx,is(x,y), where *G*_*x*,*i*_ ≠ ∅

In the formulations in Table 1, *P*_*x*,*i*_ represents a prediction in the form of a numeric score representing how interested target user x would be in a specific item i based on their similarities to and ratings by his/her K neighbors, ***G***_***x*,*i***_ represents a set of users who are neighbors of user x and have rated item i, and $\bar{r_{x}}$ denotes the average rating of the users. In the next subsection, TOPSIS is introduced as a useful multi-attribute decision-making (MADM) technique for the ranking and selection of a number of alternatives based on several criteria.

### Multi-attribute decision-making method

As mentioned in the preceding section, most studies on improving the accuracy of CF have focused on improving the similarity measure, even though the prediction score model is of similar importance [53]. In memory-based CF, after locating a target user’s neighbors, the system collects their items and predicts the rating scores that the target user would apply to them; the items are then ranked and recommended according to these predicted scores. Clearly, the prediction algorithm plays an important role in this process. As a replacement for prediction, we propose the use of TOPSIS as a useful MADM method for evaluating and ranking items.

The numerous alternatives people face online can render the decision-making process difficult, particularly when called upon to rank or choose the best alternative from a set of available items. In general, multiple criteria are used to evaluate sets of alternatives. For example, the main criteria in purchasing a car include cost, safety, comfort, and fuel consumption. Multi-criteria decision making (MCDM), one of the better-known approaches for deciding among alternatives, can be applied when the decision maker’s preferences must be taken into account. The literature divides MCDM problems into two basic approaches [70]: multi-objective decision making (MODM) and multi-attribute decision making (MADM).

MADM problems are distinguished from MODM problems by the number of predetermined decision alternatives. In MADM, decision problems are subjected to a number of decision criteria to produce rankings of multiple alternatives according to their attributes. This primarily involves gathering information and evaluating it against additional information provided by the decision maker, resulting in a decision matrix that is used to determine the final ranking of alternatives [71].

Hwang and Yoon [72] describe several MADM methods, including the TOPSIS. Originally presented by Yoon and Hwang [73], TOPSIS is a practical method for ranking and selecting several externally determined alternatives through the use of distance measures [74]. The primary advantages of TOPSIS include its ability to quickly identify the best alternative [75] and comparable or superior performance to that of simple additive weighting and analytic hierarchy processes, respectively [76]. A limited number of simple inputs (i.e., the weights associated with the respective criteria [76]) are required of decision-makers, and the output of the process is easy to understand. The underlying principle of TOPSIS is that the best alternative is that located closest to the ideal solution and furthest from the negative ideal solution [77].

The TOPSIS technique is implemented in several computational steps, which are outlined as follows:

- determine the decision alternatives;
- identify the criteria (attributes) that are related to the decision problem;
- construct a decision matrix containing *m* alternatives associated with *n* attributes (or criteria);
- normalize the raw scores to construct a precedence score, or normalized decision, matrix. The scores in the normalized matrix should be transformed into a normalized scale;
- construct a weighted normalized decision matrix in which each attribute is given a specific weight to reflect how important it is to the overall decision;
- identify the ideal and negative-ideal solutions;
- calculate the separation measure as an n-dimensional Euclidean distance between alternatives;
- calculate the relative closeness of each alternative to the ideal solution;
- create a ranking of alternatives based on the maximization of the relative closeness measures in the preceding step.

These steps will be explained in more detail in the next section.

## Proposed memory-based CF using TOPSIS

**Fig 1:**
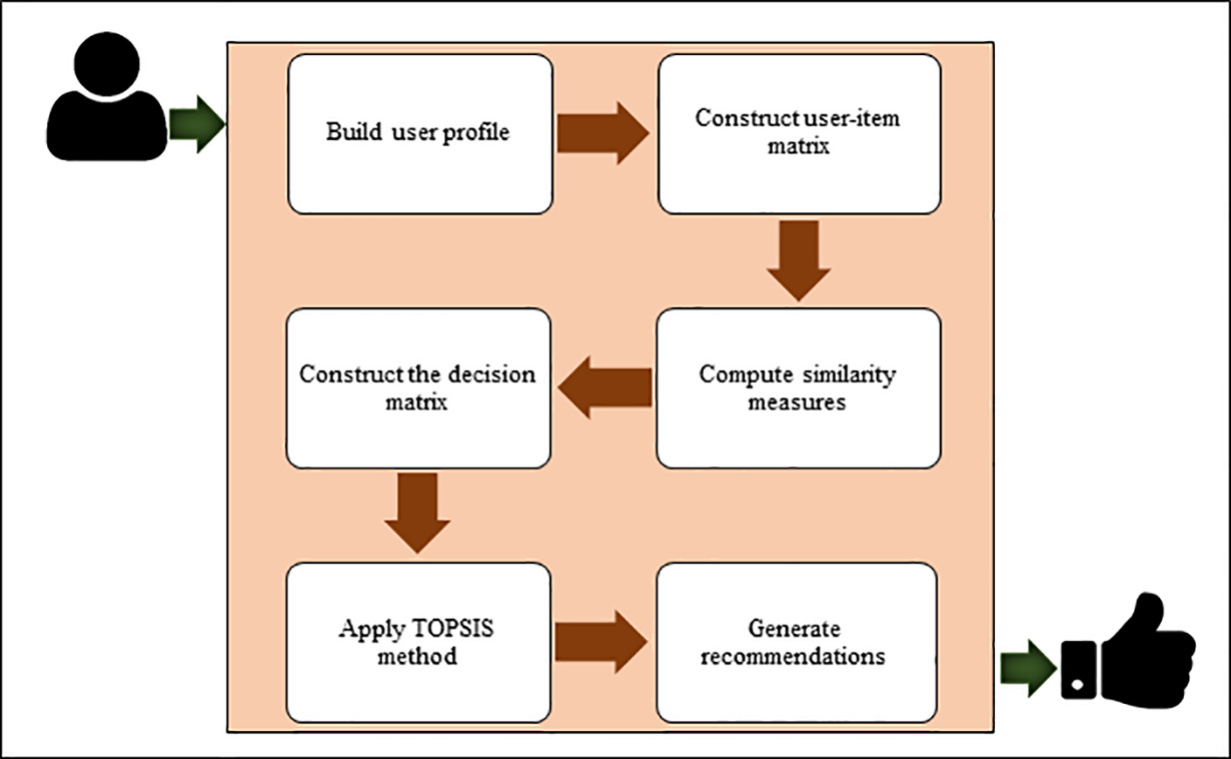
Architecture of the proposed memory-based CF method.

The proposed technique involves the application of TOPSIS to the recommendation of sets of items that might be of interest to a user. This is implemented over several main phases, as shown by the architecture in Fig 1.

The phases of the proposed method are summarized as follows:

- *Build user profile*: The system gathers feedback from a target user to build his/her preference profile. Such preferences are conventionally associated with a scale of values representing the degree of user preference for an item, e.g., one-to-five stars or one-to-ten points. A user *x* rating movie *a* with a “five” score and movie *b* with a “three” score could therefore be seen to prefer *a* over *b*.
- *Construct user-item matrix*: Data relating to users and items in the system are entered into a user-item matrix as a collection of numerical ratings.
- *Compute similarity measures*: The similarities among users are calculated using several common CF baseline methods (e.g., PCC, CPCC, SPCC, Cos, MSD, JMSD, NHSM). Following this, the top-*K* users with the strongest correlations in terms of similarity with the target user are used to form his/her neighborhood.
- *Construct the decision matrix*: The attributes of the *K*-nearest users are collected and used to populate a matrix of alternatives comprising items that have been rated by these users but not yet chosen by the target user. Items that have not been rated are assigned values based on a default vote [58].
- *Apply TOPSIS method*: The TOPSIS method [71, 73] is then applied to evaluate and rank all of the alternative items. As discussed in the next section, TOPSIS identifies the best alternative as the one with the shortest and furthest distances from the ideal and negative-ideal solutions, respectively. TOPSIS allows the best alternative to be identified quickly [75], is easy to implement, requires only a limited number of inputs from decision-makers, and produces easily understandable output. The only input parameters are the weight values associated with the criteria [76]. This main phase of the process will be explained in detail in the following subsection.
- *Generate recommendations*: As described above, the output produced by TOPSIS is a list of sorted alternatives (candidate items) ranked according to an importance measurement based on several criteria (*K*-neighbors). In the final phase of the recommendation process, the Top-M items are selected and presented to the target user as a set of item suggestions.

### TOPSIS technique

In the proposed method, the TOPSIS technique is used in place of prediction ratings to evaluate and rank candidate items and produce a sorted list of item recommendations in terms of their predicted preference. An essential input to this procedure is the list of *K*-neighbors and their items, ratings, and similarity weights with respect to the target user. TOPSIS converts this selection and ranking problem into a decision matrix X with *m* alternatives (rows) and *n* criteria (columns) corresponding to the candidate items and *K*-neighbors, respectively. In X, each entry *x*_*i*,*j*_ represents the numerical outcome of the *j*^*th*^ alternative with respect to the *i*^*th*^ criterion, i.e., the rating value applied by user *i* to item *j*. To avoid division by zero during execution, missing ratings are represented by an average for each user. Because the criteria cannot be assumed to have equal importance, a set of weighting parameters provided by the decision-maker is associated with the criteria. These weights are then compared to those of the decision-maker neighbors to obtain the set of *K*-neighbors.

Before examining the functioning of TOPSIS in detail, we define the sets used in the analysis:

- *A* is the set of candidate items representing the alternatives *A* = {*a*_1_,*a*_2_,…,*a*_*j*_,…,*a*_*m*−1_,*a*_*m*_}, where *j* = 1,2,…,*m* and *m* is the total number of candidate items.
- *C* is the set of neighbors representing the various criteria *C* = {*c*_1_,*c*_2_,…,*c*_*i*_,…,*c*_*n*−1_,*c*_*n*_}, where *i* = 1,2,…,*n* and *n* denotes the number of criteria (*K*-neighbors).
- *X* is the set of ratings *X* = {*x*_*j*,*i*_|*j* = 1,…,*m*; *i* = 1,…,*n*}, where *x*_*j*,*i*_ is the rating value of the *j*^*th*^ alternative/item with respect to the *i*^*th*^ criterion/neighbor user.
- *W* is the set of weights *W* = {*w*_1_,*w*_2_,…,*w*_*i*_,…,*w*_*n*−1_,*w*_*n*_|*i* = 1,2,…,*n*}, where *w*_*i*_ is the weight of the *i*^*th*^ criterion/neighbor (i.e., the similarity value between the *i*^*th*^ neighbor and the target user).

A decision matrix *X* containing *m* alternatives associated with *n* criteria is represented in Table 2.

**Table 2:** Conceptual decision matrix X.

			Neighbors						
**X =**			***c***_**1**_	***c***_**2**_	**⋯**	_***ci***_	**⋯**	***c***_***n*−1**_	***c***_***n***_
	**Candidate Items**	***a***_**1**_	***x***_**1,1**_	***x***_**1,2**_	**⋯**	***x***_**1,*i***_	**⋯**	***x***_**1,*n*−1**_	***x***_**1,*n***_
		***a***_**2**_	***x***_**2,1**_	***x***_**2,2**_	**⋯**	***x***_**2,*i***_	**⋯**	***x***_**2,*n*−1**_	***x***_**2,*n***_
		**⋮**	**⋮**	**⋮**	**⋱**	**⋮**	**⋱**	**⋮**	**⋮**
		***a***_***j***_	***x***_***j*,1**_	***x***_***j*,2**_	**⋯**	***x***_***j*,*i***_	**⋯**	***x***_***j*,*n*−1**_	***x***_***j*,*n***_
		**⋮**	**⋮**	**⋮**	**⋱**	**⋮**	**⋱**	**⋮**	**⋮**
		***a***_***m*−1**_	***x***_***m*−1,1**_	***x***_***m*−1,2**_	**⋯**	***x***_***m*−1,*i***_	**⋯**	***x***_***m*−1,*n*−1**_	***x***_***n*−1,*n***_
		***a***_***m***_	***x***_***m*,1**_	***x***_***m*,2**_	**⋯**	***x***_***m*,*i***_	***x***_***m*,1**_	***x***_***m*,*n*−1**_	***x***_***n*,*n***_

The steps in the TOPSIS method are described as follows.

**Step 1**: *Construct a normalized decision matrix*

Some users prefer to provide high ratings, even for items they do not like very much, whereas others will give low ratings to items they like. To account and adjust for such rating disparities and irregularities, it is necessary to normalize the decision matrix. This can be done through distributive normalization, in which the rating values in each column are divided by the square root of the sum of each squared alternative in the column. The elements *r*_*j*,*i*_ of the normalized decision matrix R are therefore given by:

$$r_{j,i}=\frac{x_{j,i}}{\sqrt{\sum_{j=1}^{m}{x_{j,i}}^{2}}},j=1\ldots m;i=1\ldots n.$$ (16)

The results of applying Eq (16) to matrix X to produce the normalized matrix R are presented in Table 3.

**Table 3:** Conceptual normalized decision matrix R.

			Neighbors						
**R =**			***c***_**1**_	***C***_**2**_	**⋯**	***c***_***i***_	**⋯**	***c***_***n−*1**_	***c***_***n***_
	**Candidate Items**	***a***_**1**_	***r***_**1,1**_	***r***_**1,2**_	**⋯**	***r***_**1,*i***_	**⋯**	***r***_**1,*n*−1**_	***r***_**1,*n***_
		***a***_**2**_	***r***_**2,1**_	***r***_**2,2**_	**⋯**	***r***_**2,*i***_	**⋯**	***r***_**2,*n*−1**_	***r***_**2,*n***_
		**⋮**	**⋮**	**⋮**	**⋱**	**⋮**	**⋱**	**⋮**	**⋮**
		***a***_***j***_	***r***_***j*,1**_	***r***_***j*,2**_	**⋯**	***r***_***j*,*i***_	**⋯**	***r***_***j*,*n*−1**_	***r***_***j*,*n***_
		**⋮**	**⋮**	**⋮**	**⋱**	**⋮**	**⋱**	**⋮**	**⋮**
		***a***_***m*−1**_	***r***_***m*−1,1**_	***r***_***m*−1,2**_	**⋯**	***r***_***m−*1,*i***_	**⋯**	***r***_***m−*1,*n*−1**_	***r***_***n−*1,*n***_
		***a***_***m***_	***r***_***m*,1**_	***r***_***m*,2**_	**⋯**	***r***_***m*,*i***_	***r***_***m*,1**_	***r***_***m*,*n*−1**_	***r***_***n*,*n***_

**Step 2**: *Construct the weighted normalized decision matrix*

To take the weights W provided by the decision-maker into account, a weighted normalized decision matrix V is given by multiplying the normalized values *r*_*j*,*i*_ by their corresponding weights *w*_*i*_. In the proposed method, the similarity weights of the target user with respect to his/her neighbors are used to develop the user’s weight criteria. For example, for a target user *u* who has *k* neighbors (with *n* criteria), the similarity weights *s*_*u*_ = {*s*_*u*,1_,*s*_*u*,2_,…,*s*_*u*,*i*_,…,*s*_*u*,*n*−1_,*s*_*u*,*n*_|,*i* = 1,2,…,*n*}, where *s*_*i*,*k*_ denotes the similarity value between *u* and the *i*^*th*^ neighbor, are used to populate the set of weights *w*_*i*_. The weighted normalized decision matrix V is then obtained as follows:

$$v_{j,i}=r_{j,i}*w_{i},j=1\ldots m;\;i=1\ldots n.$$ (17)

Table 4 presents a weighted normalized decision matrix V obtained by applying Eq (17) to the normalized decision matrix R.

**Table 4:** Conceptual weighted normalized decision matrix V.

			Neighbors						
**V =**			***c***_**1**_	***c***_**2**_	**⋯**	***c***_***i***_	**⋯**	***c***_***n−*1**_	***c***_***n***_
	**Candidate Items**	***a***_**1**_	***v***_**1,1**_	***v***_**1,2**_	**⋯**	***v***_**1,*i***_	**⋯**	***v***_**1,*n*−1**_	***v***_**1,*n***_
		***a***_**2**_	***v***_**2,1**_	***v***_**2,2**_	**⋯**	***v***_**2,*i***_	**⋯**	***v***_**2,*n*−1**_	***v***_**2,*n***_
		**⋮**	**⋮**	**⋮**	**⋱**	**⋮**	**⋱**	**⋮**	**⋮**
		***a***_***j***_	***v***_***j*,1**_	***v***_***j*,2**_	**⋯**	***v***_***j*,*i***_	**⋯**	***v***_***j*,*n*−1**_	***v***_***j*,*n***_
		**⋮**	**⋮**	**⋮**	**⋱**	**⋮**	**⋱**	**⋮**	**⋮**
		***a***_***m*−1**_	***v***_***m*−1,1**_	***v***_***m*−1,2**_	**⋯**	***v***_***m−*1,*i***_	**⋯**	***v***_***m−*1,*n*−1**_	***v***_***n−*1,*n***_
		***a***_***m***_	***v***_***m*,1**_	***v***_***m*,2**_	**⋯**	***v***_***m*,*i***_	***v***_***m*,1**_	***v***_***m*,*n*−1**_	***v***_***n*,*n***_

**Step 3**: *Determine positive and negative ideal solutions*

The best and worst evaluation alternatives for each criterion in the normalized decision matrix V are then identified and used to represent the ideal and negative-ideal solutions, respectively.

For a set of positive attributes or criteria *I*_1_ associated with benefit (more is better) and a set of negative attributes or criteria *I*_2_ associated with cost (less is better), the positive- and negative-ideal solutions can be defined as follows:

Ideal solution:

$$A^{*}=\{{v_{1}}^{*},\ldots ,{v_{i}}^{*},\ldots ,{v_{n}}^{*}\},where\;{v_{i}}^{*}=\{\max\left(v_{j,i}\right)ifc_{i}\in I_{1};\min\left(v_{j,i}\right)ifc_{i}\in I_{2}\},$$ (18)

Negative-ideal solution:

$$A^{\prime }=\left\{v_{1}{}^{\prime },\ldots ,v_{i}{}^{\prime },\ldots ,v_{n}{}^{\prime }\right\},where\;v_{i}{}^{\prime }=\left\{min\left(v_{j,i}\right)ifc_{i}\in I_{1};max\left(v_{j,i}\right)ifc_{i}\in I_{2}\right\}$$ (19)

The alternatives A* and *A*′ represent the most-favored (ideal solution) and least-favored (negative-ideal solution) options, respectively.

**Step 4**: *Calculate the separation measure*

The distance from each alternative to the ideal and negative-ideal solutions for all alternatives can be calculated using the Euclidean distance measurement. The distance of each alternative from the ideal is given by:

$${S_{j}}^{*}=\sqrt[{2}]{\sum_{i=1}^{n}{{(v}_{j,i}–{v_{j}}^{*})}^{2}},j=1,2,\ldots ,m.$$ (20)

Similarly, the distance of each alternative from the negative-ideal is given by:

$${S_{j}}^{\prime }=\sqrt[{2}]{\sum_{i=1}^{n}{{(v}_{j,i}–{v_{j}}^{\prime })}^{2}},j=1,2,\ldots ,m.$$ (21)

Table 5 gives an example of a separation matrix (V′).

**Table 5:** Conceptual separation matrix V′.

**V′ =**			S*	***S*′**
	**Candidate items**	***a***_**1**_	***S***_1_*	***S***_**1**_**′**
		***a***_**2**_	***S***_2_*	***S***_**2**_**′**
		**⋮**	**⋮**	**⋮**
		***a***_***j***_	***S***_j_*	***S***_**j**_′
		**⋮**	**⋮**	**⋮**
		***a***_***m*−1**_	***S***_*m*−1_^*^	***S***_***m*−1**_**′**
		***a***_***m***_	***S***_m_^*^	***S***_***m***_**′**

**Step 5**: *Calculate the relative closeness to the ideal solution*

The degree of closeness of each alternative to the ideal solution A* is calculated as

$${C^{*}}_{j}={S_{j}}^{\prime }/{{(S}_{j}}^{*}+{S_{j}}^{\prime }),0<{C^{*}}_{j}<1,j=1,2,...,m.$$ (22)

**Fig 2:**
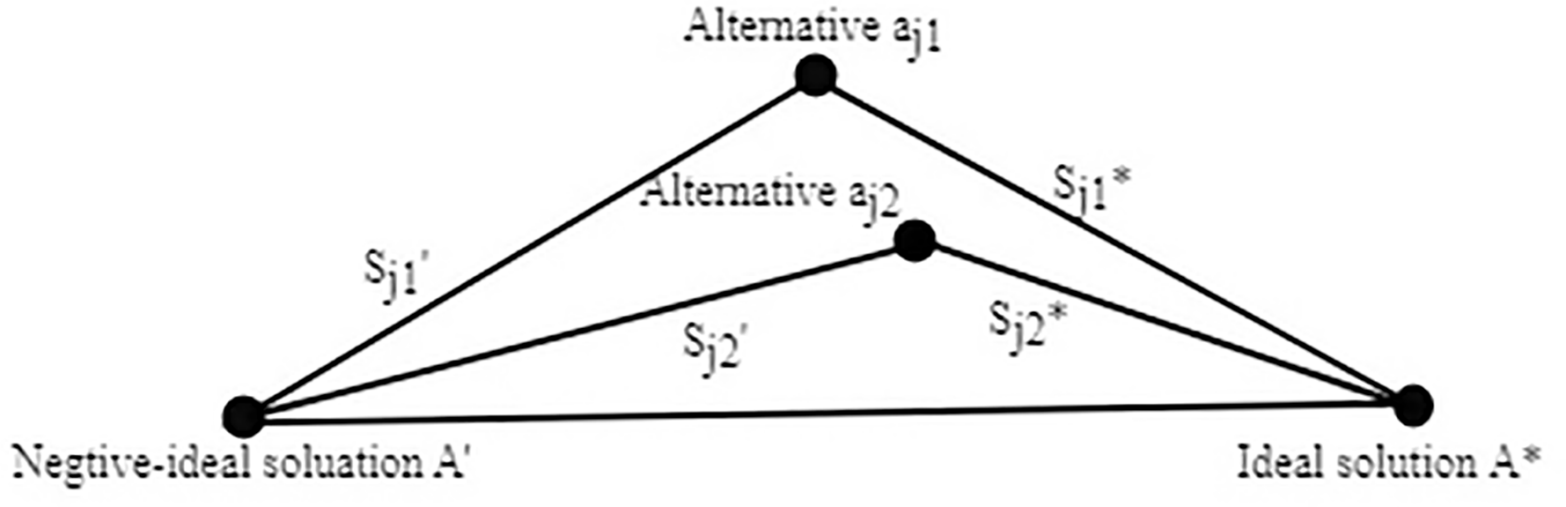
Euclidean distances to the ideal and negative-ideal solutions.

The relative closeness rating ranges between zero and one; these extremes represent, respectively, the least- and most-favored alternatives. To elaborate, if the distance of alternative *a*_*j*_ from the ideal solution A* is smaller than its distance from the negative-ideal *A*′, then C*_j_ will be closer to one than to zero, and vice versa, as shown in Fig 2.

**Step 6**: Ranking the alternatives in order according to C*_j_

To produce an outcome in the form of a sorted list of alternatives, TOPSIS determines a preference order by arranging the alternatives in descending order of closeness degree C*_j_.

## Experimental setup and results

### Datasets

Experiments were performed using four widely used and publicly available datasets, namely MovieLens 100K and 1M, HetRec2011, and FilmTrust. The MovieLens [78] 100K and 1M datasets, collected by the GroupLens research group at the University of Minnesota (http://grouplens.org/datasets/MovieLens/), are often used by CF systems [11, 51]. For this study, they were used to evaluate the performance of the proposed technique in combination with several common memory-based CF methods. The MovieLens 100K dataset, which was initially released in April 1998, includes 100,000 ratings of 1,682 movies provided by 943 users. It only captures users who have rated 20 or more movies. The 1M MovieLens dataset, which was initially released in February 2003, contains 1,000,209 ratings of approximately 3,900 movies from 6,040 users. In both datasets, the ratings are given on a scale of one to five stars with a one-star granularity. The sparsity values of 100k and 1M are 93.7 and 95.8%, respectively.

The HetRec2011-MovieLens dataset is an extension of a dataset published by GroupLens (http://grouplens.org/). This dataset was released in the framework of the 2nd International Workshop on Information Heterogeneity and Fusion in Recommender Systems [79]. HetRec2011-MovieLens consists of 855,598 ratings provided by 2,113 users on 10,197 movies and has 96.03% sparsity. The FilmTrust dataset (https://www.librec.net/datasets.html) contains 35,497 ratings provided by 1,986 users on 2,071 items and has 98.86% sparsity [80].

### Experimental process

The experimental process to evaluate the proposed method was conducted as follows:

- Each dataset was partitioned into five equally sized sets to allow the cross-validation method to be applied [81]. In five separate trials, one subset was used as the test set (20%) and the other four were combined to form a training set (80%), with the test and training set roles rotated across trials. The average result across all trials was then computed.
- Based on the user-item rating matrix, the similarity between users was calculated using PCC, CPCC, SPCC, COS, MSD, JMSD, and NHSM. A set of *K*-nearest neighbors was then formed for the results produced by each similarity method.
- The items ranked by each *K*-nearest neighbor set were collected and any items that the active user had previously selected were removed to obtain sets of candidate items.
- Decision matrices were constructed and the TOPSIS technique was applied to them to obtain sets of ranked items.
- Finally, the top M items were identified as recommendations and presented to the target user.

**Fig 3:**
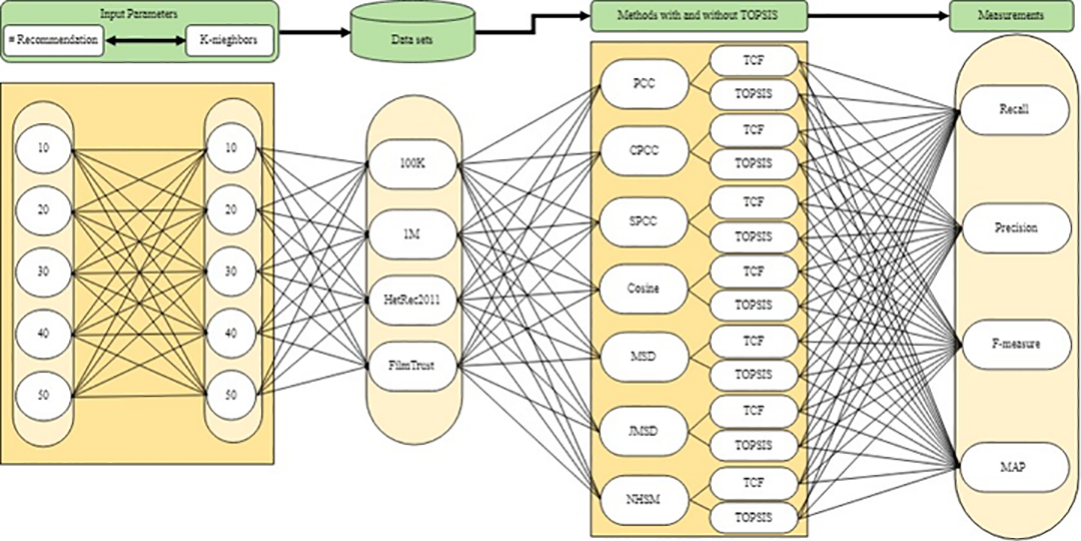
Experimental process with respect to input parameters, datasets, methods with & without TOPSIS, and measurements.

The accuracy of CF recommender systems is influenced by two parameters, namely the number K of neighbors and the size of the recommendation list. These two parameters should be fixed in the experimental process to ensure a fair comparison among algorithms [12, 82]. Hence, the experiments were executed with K values of 10, 20, 30, 40, and 50 and recommended list sizes of 10, 20, 30, 40, and 50. Fig 3 illustrates the experimental process with respect to input parameters, datasets, baseline CF methods with and without TOPSIS, and the measurements. A total of 25 experiments were conducted on each of the four datasets using all seven baseline methods with and without TOPSIS. Four metrics were selected to evaluate the proposed method. These metrics, which are described in the following subsection, are widely used to evaluate the accuracy of memory-based CF techniques.

### Evaluation metrics

The TOPSIS technique was applied as an MADM approach in conjunction with various conventional memory-based CF methods, and the results were compared with those obtained without the use of TOPSIS. The recall, precision, and F-measure [69, 83], which are widely used to evaluate the accuracy of memory-based CF [15, 37, 84, 85], were used as performance metrics. These metrics measure the accuracy of a recommender system based on the items recommended to its users. The precision is the fraction of items rated by the users in the test set and recommended by the recommender system. The precision metric represents the ratio of the recommended items to the total number of items recommended by the system, and is given by Eq (23). The recall metric is the fraction of rated items recommended by a recommender system. The recall represents the ratio of the recommended items to all of the items rated by the users in the test set, and is defined by Eq (24). The F-measure metric is the weighted mean of the precision and recall, and is given by Eq (25). Thus, the F-measure is a combined metric of precision and recall. Table 6 illustrates the recommendation confusion matrix and its relation to these metrics.

**Table 6:** Recommendation confusion matrix.

	Rated	Unrated
**Recommended**	TP	FP
**Not recommended**	FN	TN

The terms in Table 6 are defined as follows:

- **TP**, true positive: number of test samples belonging to the user *Interest* that are *Recommended*.
- **FN**, false negative: number of test samples belonging to the user *Interest* that are not *Recommended*.
- **TN**, true negative: number of test samples not belonging to the user *Interest* that are not *Recommended*.
- **FP**, false positive: number of test samples not belonging to the user *Interest* that are *Recommended*.

The precision, recall, and F-measure were computed using Eqs (23)–(25), respectively:

$$Precision=\frac{TP}{TP+FP},$$ (23)

$$Recall=\frac{TP}{TP+FN},$$ (24)

$$F-measure=\frac{2*(Precision*Recall)}{\left(Precision+Recall\right)},$$ (25)

The mean average precision (MAP) was also used to measure the accuracy of the ranking produced by each algorithm [2]. MAP computes the average of the precision scores over all recommendation sizes [86]. In this study, five recommendation sizes of 10, 20, 30, 40, and 50 were considered. Therefore, the *Precision* value of each specified index *j* (*Precision*@j) was computed separately. The MAP value was then normalized by dividing the sum of the *Precision* values for the specified indexes by the total number of specified indexes. The MAP value for *L* sets of specified indexes is calculated as:

$$MAP=\frac{\sum_{j=1}^{L}Precision@j)}{\left|L\right|},$$ (26)

where *Precision*@j represents the precision of the *j*^*th*^ specified index in the recommendation list, *j* = 10, 20, 30, 40, and 50. *L* is a set of predefined indices and |L| represents the size of the specified indexes.

### Results

To assess the accuracy of the proposed approach, the TOPSIS method was used to replace the prediction method in various memory-based CFs, which were then applied to the MovieLens 100K & 1M, HetRec20111, and FilmTrust datasets. Several trials were conducted using the cross-validation partitioning method and the results were assessed in terms of the recall, precision, F-measure, and MAP metrics. The results were used to construct bar graphs reflecting the accuracy over an averaged number of neighbors for *K* values of 10, 20, 30, 40, and 50.

**Fig 4:**
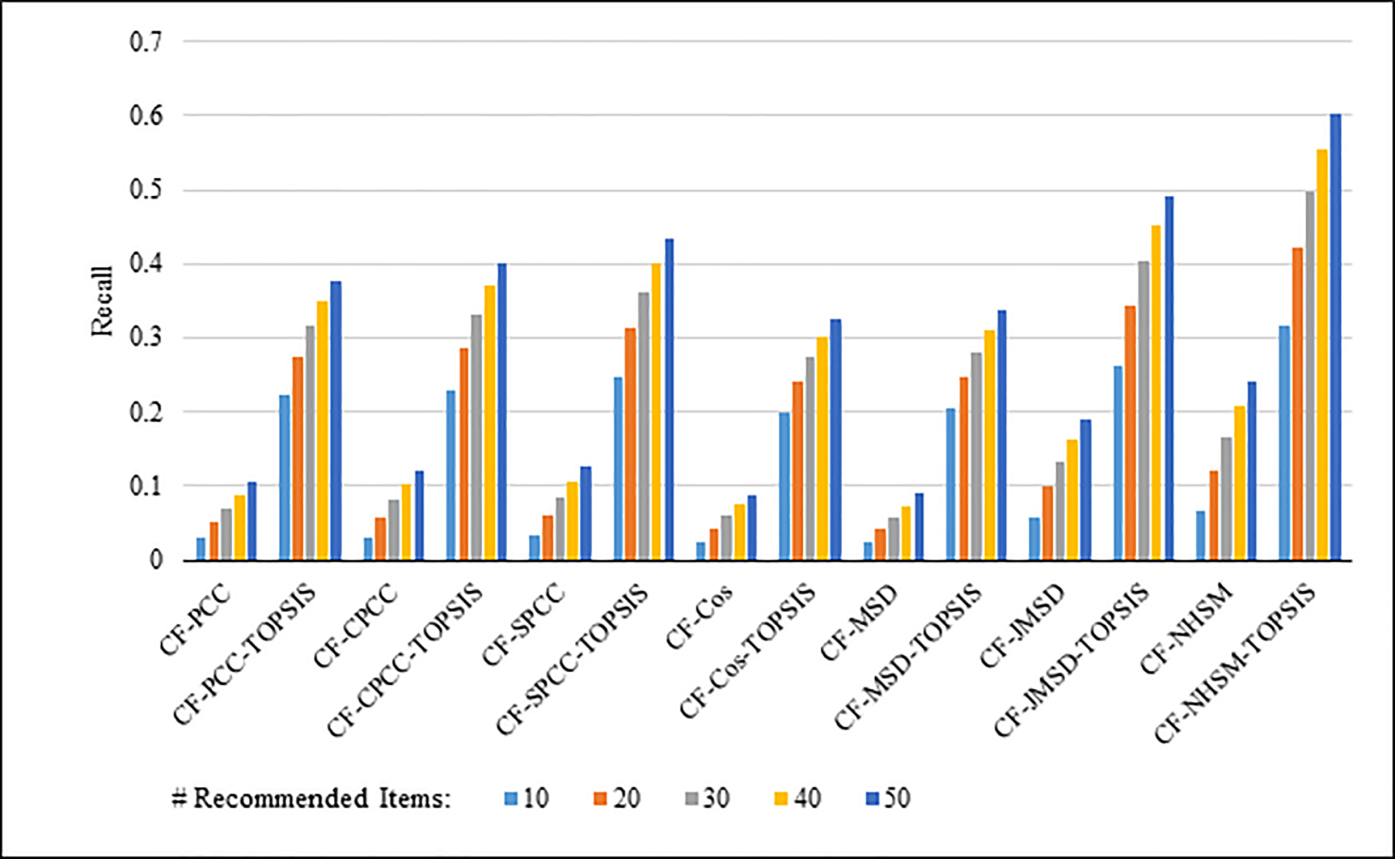
Recall measure by number of recommendations on 100K MovieLens.

**Fig 5:**
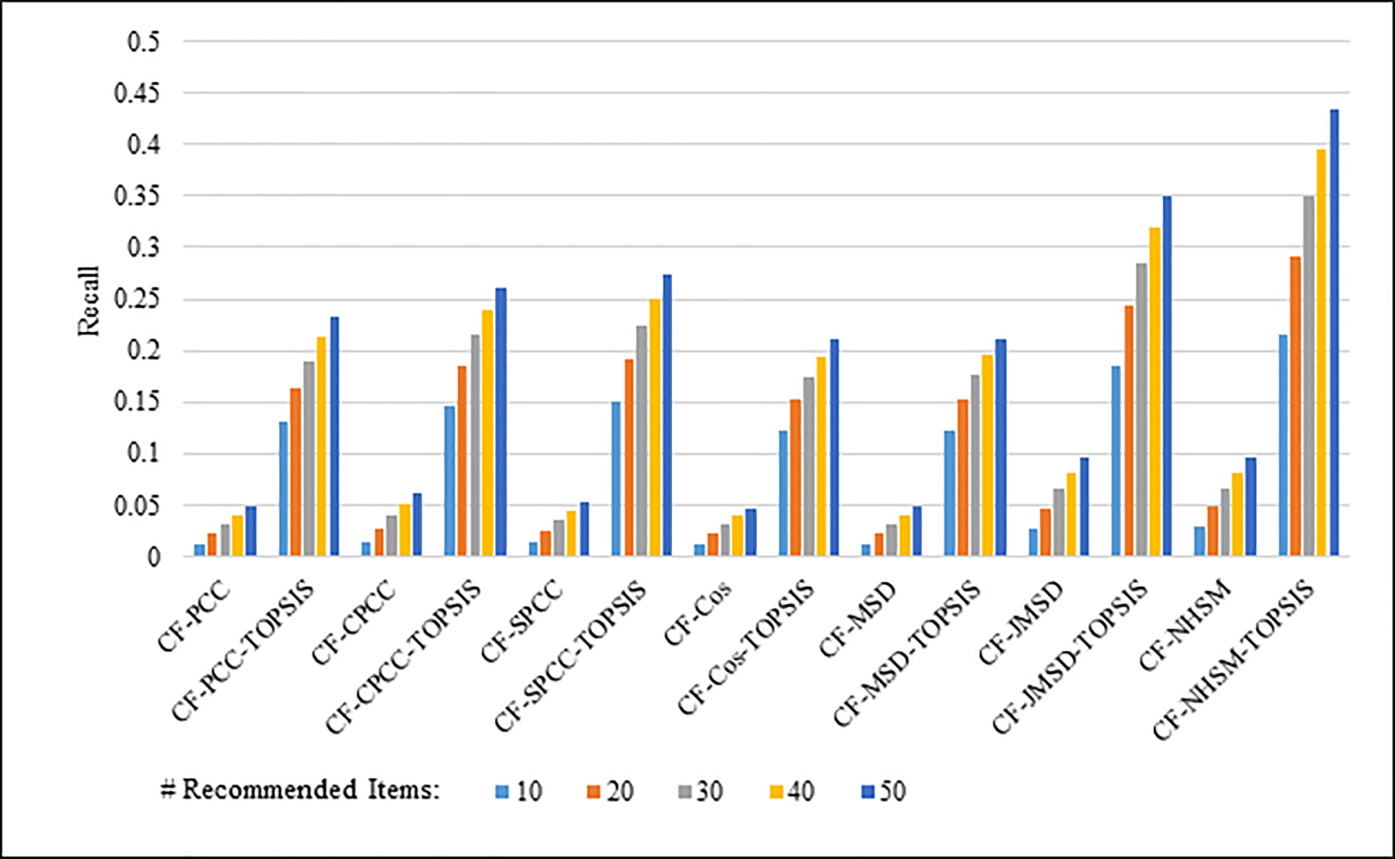
Recall measure by number of recommendations on 1M MovieLens.

**Fig 6:**
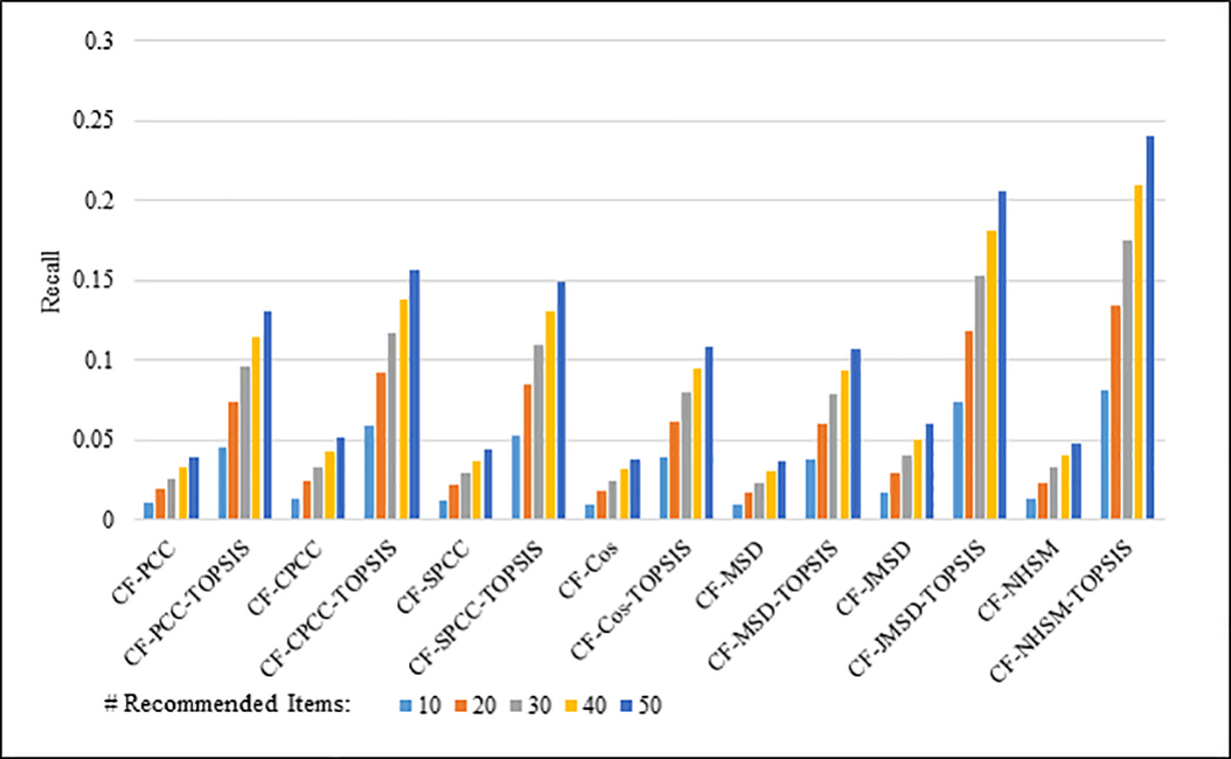
Recall measure by number of recommendations on HetRec2011.

**Fig 7:**
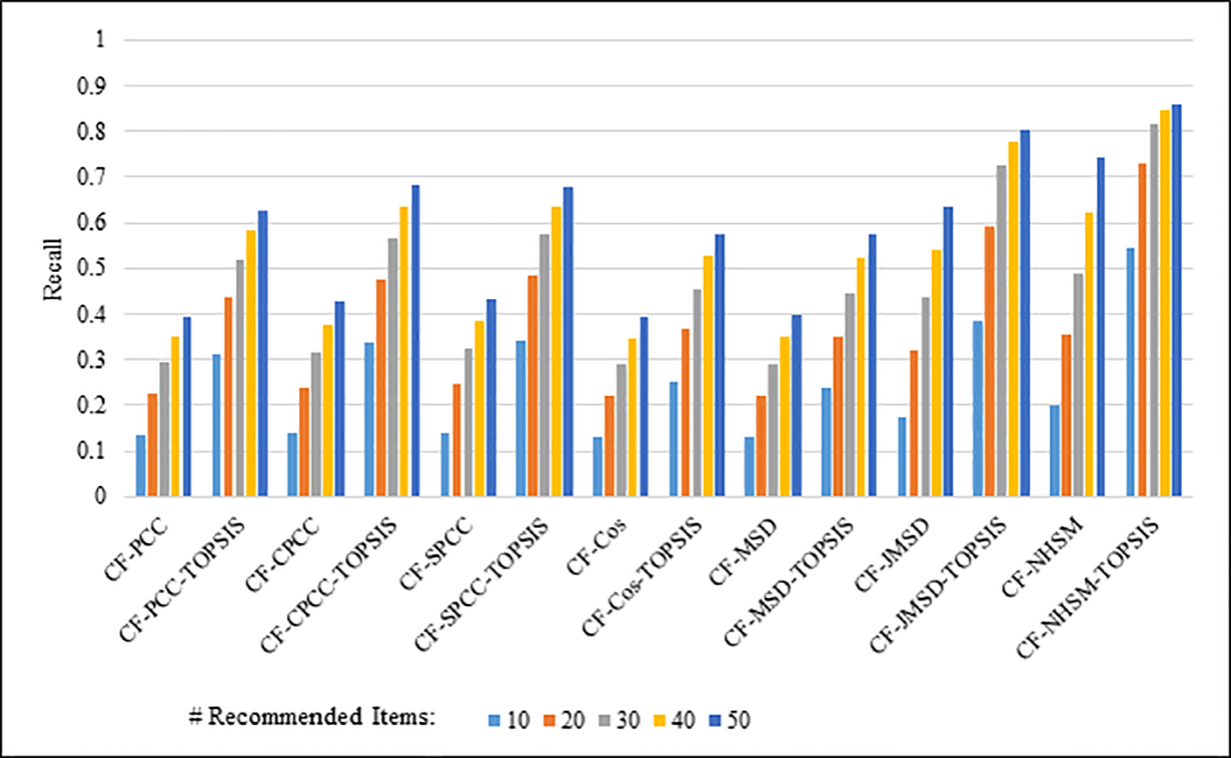
Recall measure by number of recommendations on FilmTrust.

Figs 4–7 show the recall results produced by applying PCC, CPCC, SPCC, Cos, JMSD, and NHSM with and without TOPSIS. Figs 4 and 5 show the results obtained using the 100K and 1M datasets, respectively, whereas Figs 6 and 7 show the results under cross-validation partitioning using the HetRec2011 and FilmTrust datasets, respectively. The legend shows the recommendation size in each case (10, 20, 30, 40, or 50).

The results clearly show that the use of TOPSIS produces significant improvement in terms of recall, with the TOPSIS adaptation of the NHSM CF approach producing the best results across all cases. Conversely, the Cos and MSD CF methods produce the worst recall values. In general, the recall rises with the number of recommendations. Furthermore, the results in Figs 4–6 indicate that TOPSIS increases the accuracy by a factor two when applied to the PCC, CPCC, SPCC, MSD, and Cos methods; the same figures reveal more than three-fold enhancements to JMSD and NHSM using the 100K, HetRec2011, and FilmTrust datasets. Similarly, on the 1M dataset, there are three-fold enhancements for PCC, CPCC, SPCC, MSD, and Cos, and more than four- and six-fold enhancements for JMSD and NHSM, respectively. These results indicate that the application of TOPSIS to conventional methods can significantly improve the recall performance of memory-based CF.

**Fig 8:**
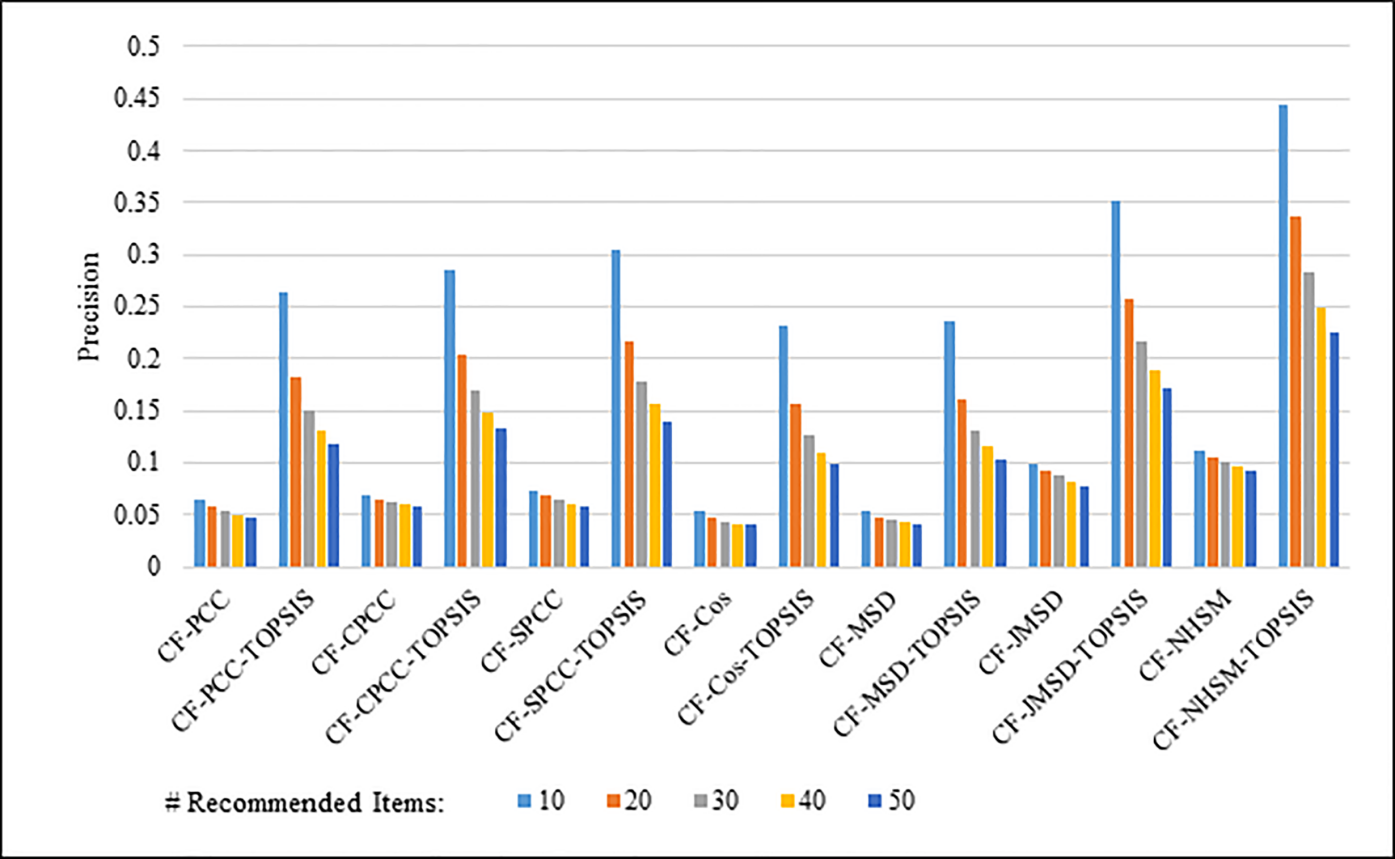
Precision measure by number of recommendations on 100K MovieLens.

**Fig 9:**
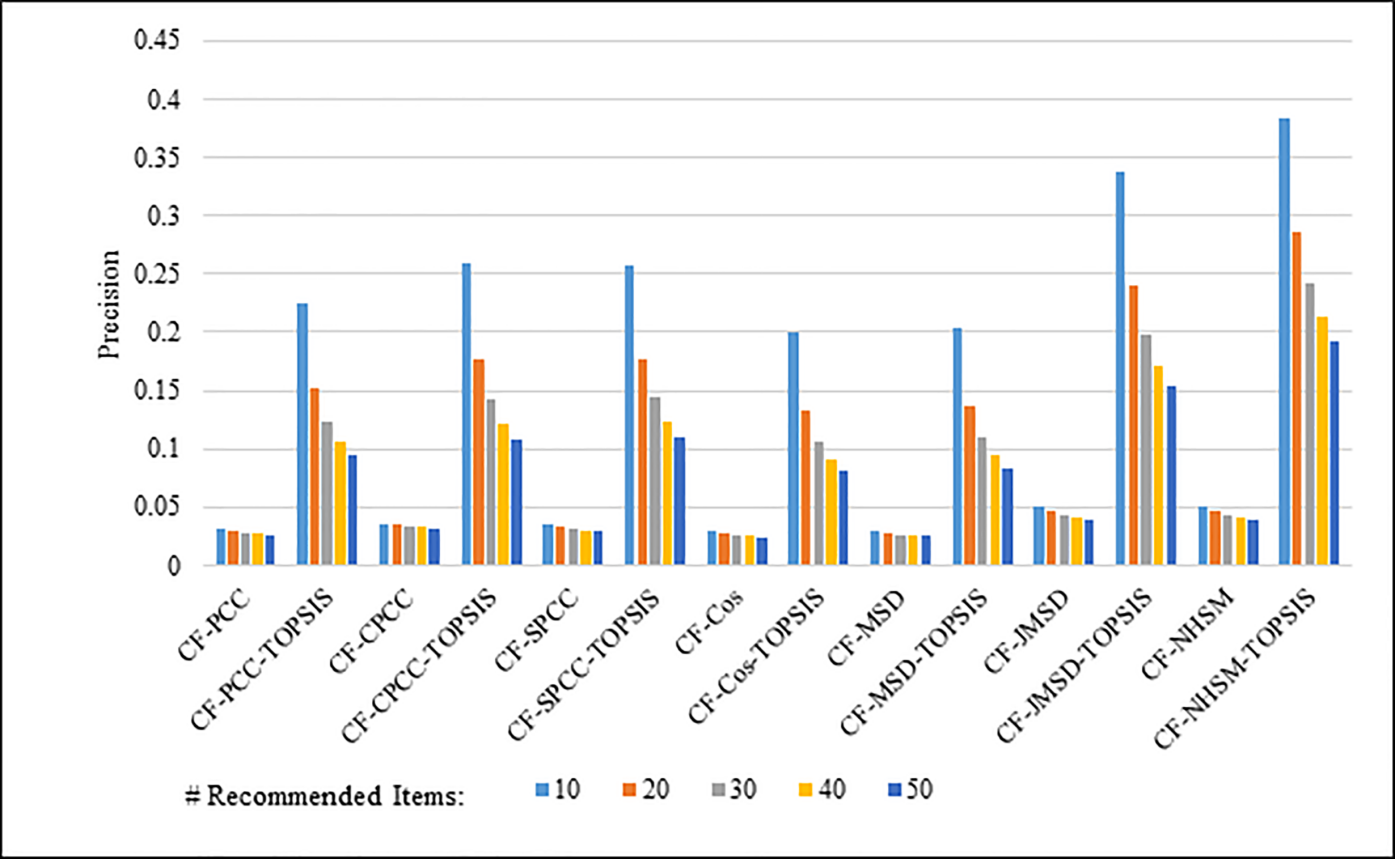
Precision measure by number of recommendations on 1M MovieLens.

**Fig 10:**
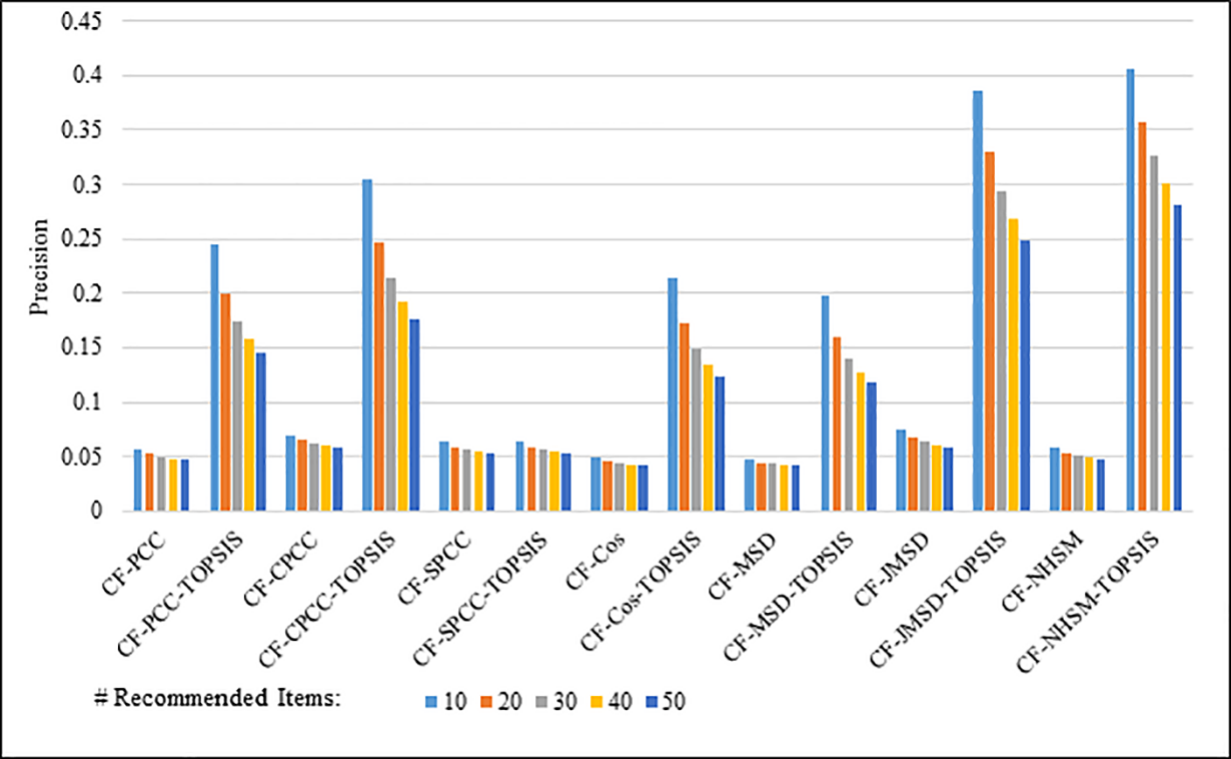
Precision measure by number of recommendations on HetRec2011.

**Fig 11:**
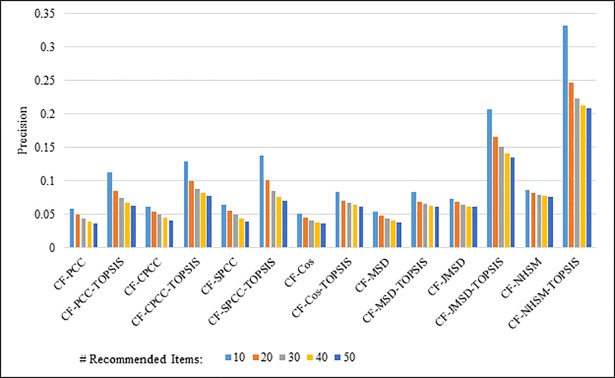
Precision measure by number of recommendations on FilmTrust.

Figs 8–11 show the precision results obtained by PCC, CPCC, SPCC, Cos, MSD, JMSD, and NHSM with and without TOPSIS. Figs 8 and 9 show the results obtained using the 100K and 1M datasets, respectively, whereas Figs 10 and 11 show the results for the HetRec2011 and FilmTrust datasets, respectively. The legend shows the recommendation size of each case (10, 20, 30, 40, or 50).

The results indicate that the application of TOPSIS produces a significant improvement in precision across all cases. It is seen that TOPSIS-enhanced NHSM has the highest precision, although the Cos and MSD methods produce results that are nearly as good. The average result with respect to the number of recommended items increases from less than 0.05 under CF-NHSM to more than 0.2 under CF-TOPSIS-NHSM, representing a four-fold increase in precision for NHSM. Contrary to the recall results, the precision gradually decreases with the number of recommendations for all methods when TOPSIS is applied. Nevertheless, the results indicate that the application of TOPSIS significantly improves the precision accuracy of memory-based CF.

**Fig 12:**
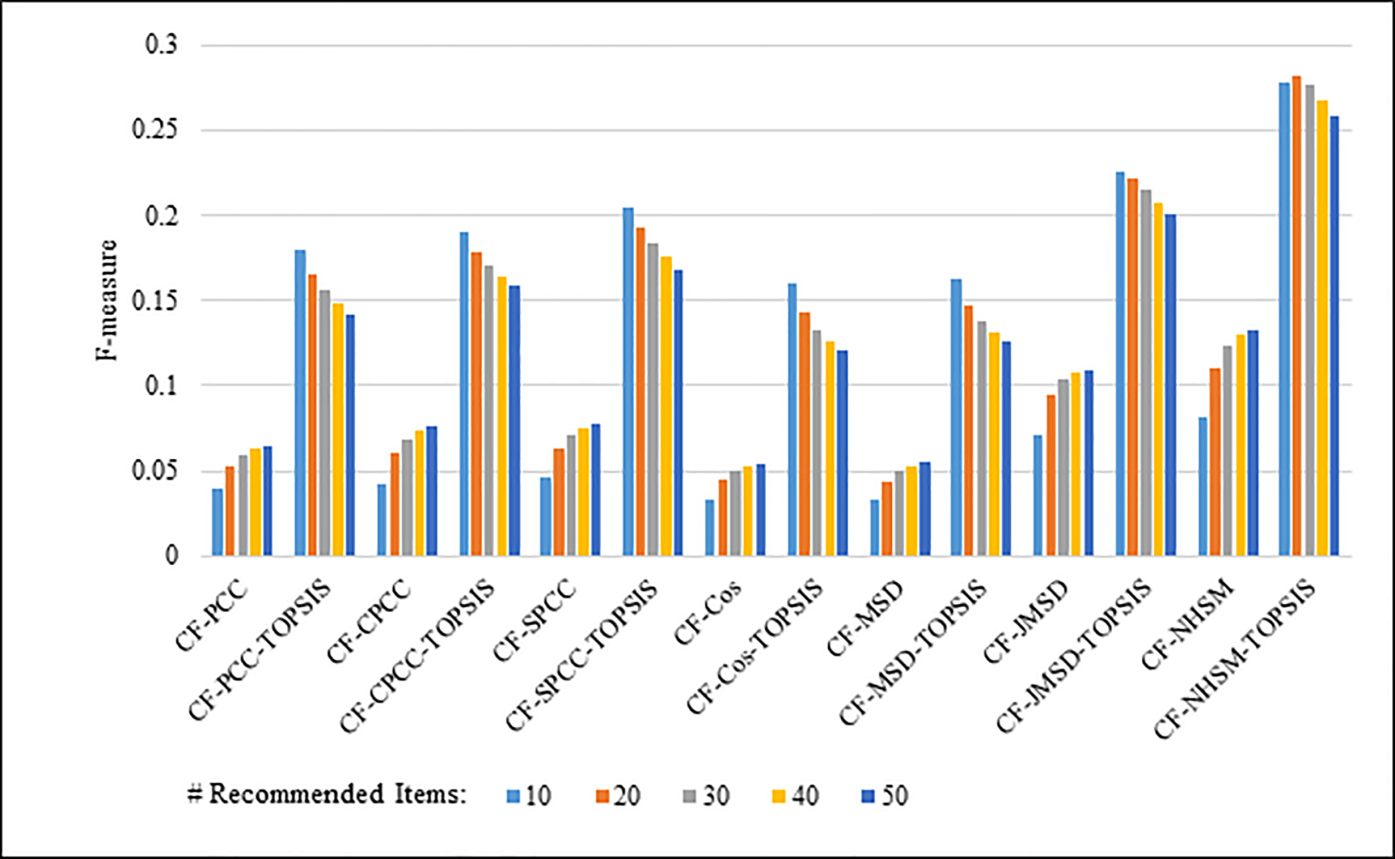
F-measure by number of recommendations on 100K MovieLens.

**Fig 13:**
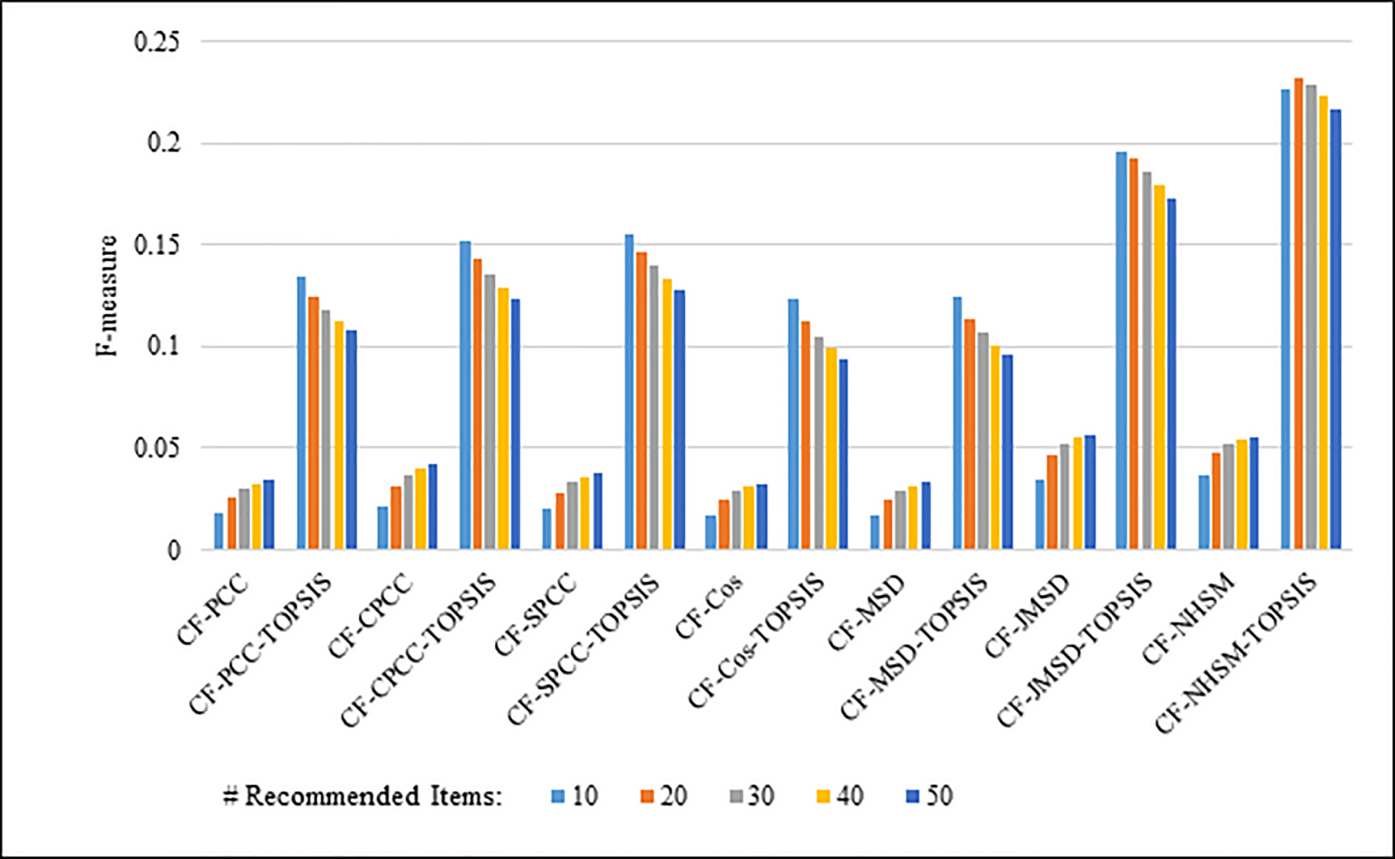
F-measure by number of recommendations on 1M MovieLens.

**Fig 14:**
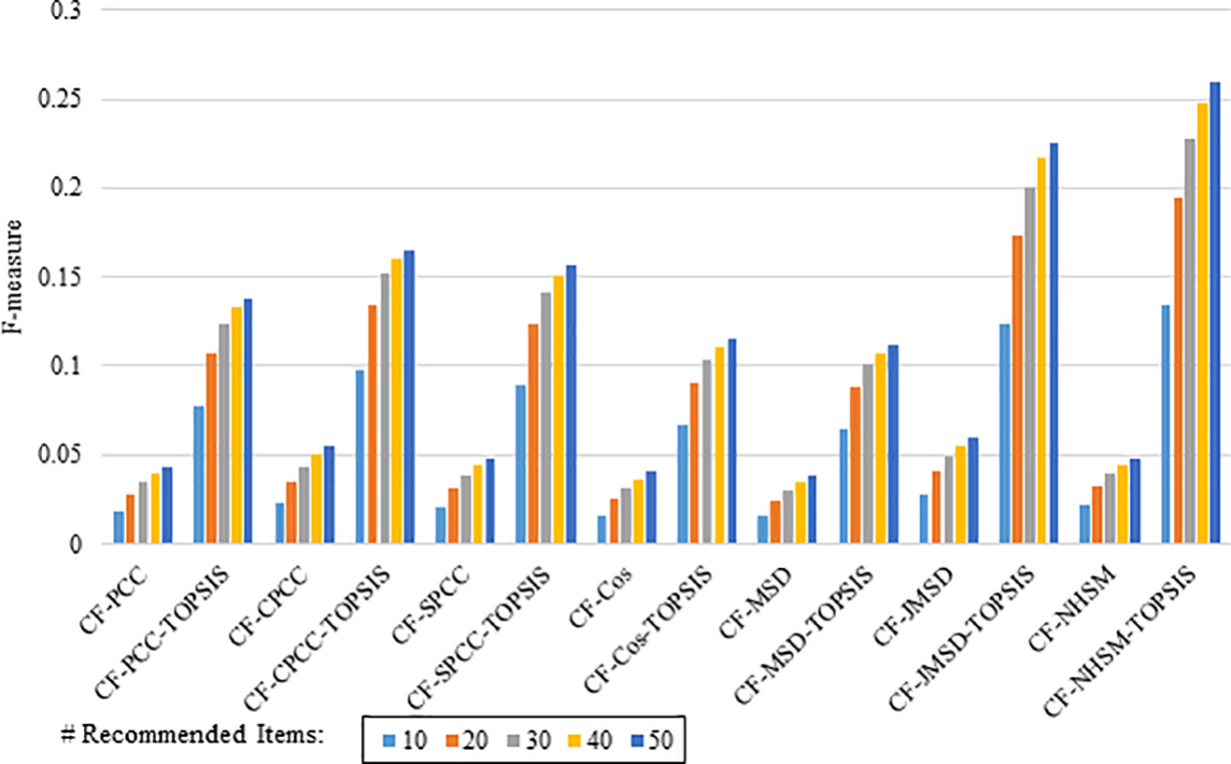
F-measure by number of recommendations on HetRec2011.

**Fig 15:**
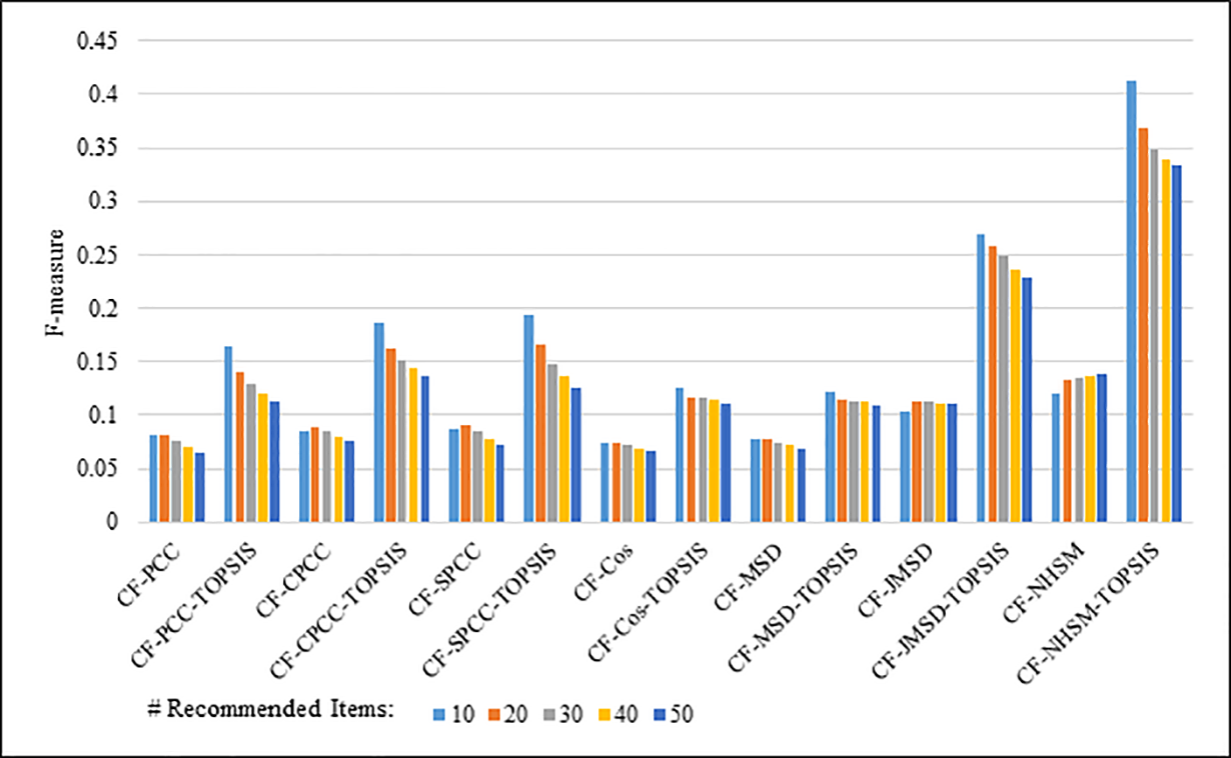
F-measure by number of recommendations on FilmTrust.

Figs 12–15 compare the F-measures produced by PCC, CPCC, SPCC, Cos, JMSD, and NHSM with and without the TOPSIS method. Figs 12 and 13 show the results obtained using the 100K and 1M MovieLens datasets, respectively, whereas Figs 14 and 15 show the F-measure results using the HetRec2011 and FilmTrust datasets, respectively. The legend denotes the different recommendation sizes of 10, 20, 30, 40, or 50.

As with the other two metrics, all methods show a notable enhancement in their F-measure results with the application of TOPSIS. In general, the F-measure decreases slightly as the number of recommendations increases. It is again seen that the TOPSIS-enhanced NHSM method produces the best results across all cases, with an improvement of approximately 50% and 75% obtained through the application of TOPSIS to (PCC, CPCC, SPCC, Cos, and JMSD) and NHSM, respectively. These results reinforce the preceding results and indicate that the application of TOPSIS significantly improves both the precision and recall of memory-based CF.

**Fig 16:**
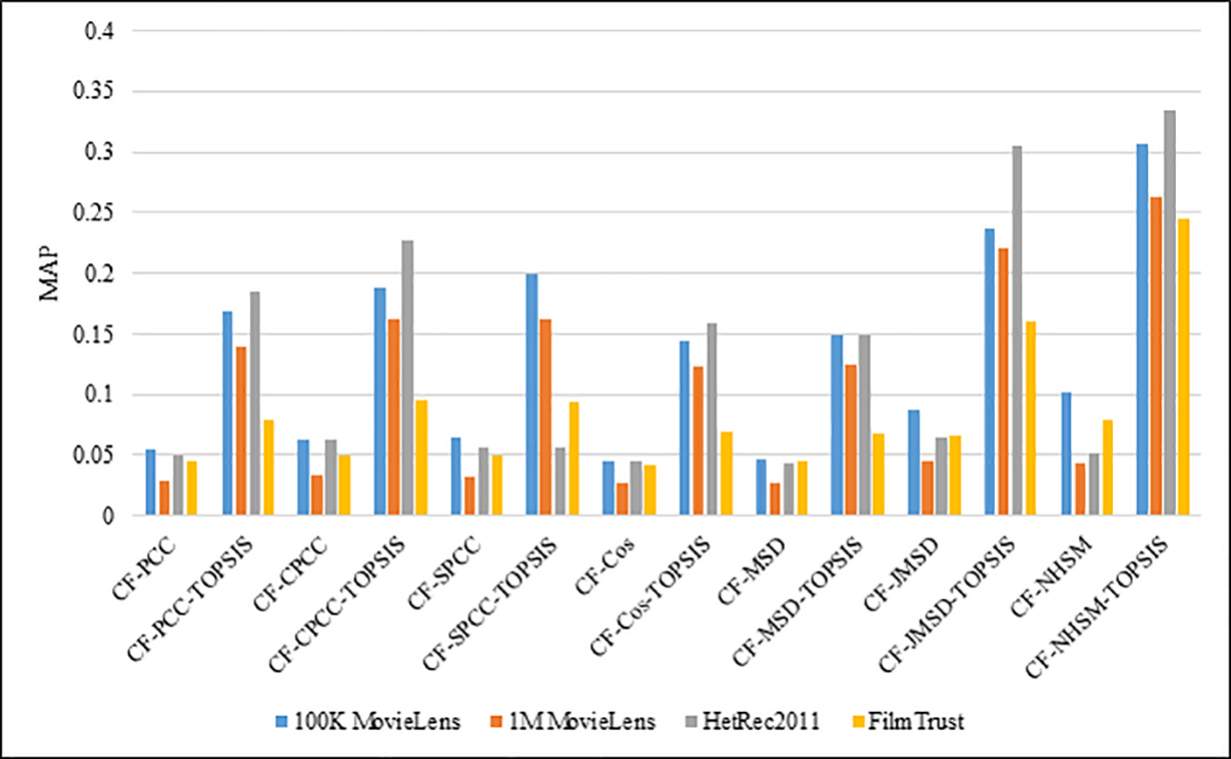
Comparison of MAP for all methods using all datasets.

Fig 16 shows the MAP results with respect to the recommendation list size for the PCC, CPCC, SPCC, Cos, JMSD, and NHSM methods with and without TOPSIS. The legend denotes the different data sets (100K & 1M MovieLens, HetRec2011, and FilmTrust).

The results clearly show that the use of TOPSIS produces a significant improvement in terms of MAP, with the TOPSIS adaptation of the NHSM CF approach producing the best results across all datasets. Conversely, the Cos and MSD CF methods produce the worst results. In terms of data sets, the MAP values using 100K MovieLens and HetRec2001 are better than with the other data sets (1M MovieLens and FilmTrust). The worst scores were obtained when applying the FilmTrust data set to all methods based on TOPSIS, except for SPCC, which performed worst using the HeRec2011 data set. Overall, the results indicate that the methods based on TOPSIS more than double the accuracy of PCC, CPCC, SPCC, MSD, and Cos, and produce three-fold enhancements in JMSD and NHSM. These results indicate that the application of TOPSIS to conventional methods can significantly improve the MAP accuracy of memory-based CF.

Generally, the RS does not guarantee that the suggested items will be relevant to the preferences of the target user, but may encourage users to find useful or interesting items. Therefore, the accuracy of the RS is affected by the user’s subsequent selection from the list of recommendations. For instance, if the recommendation list contains 10 items and the user selects just four, then the accuracy will be negatively affected by the user disregarding the other six items. Thus, in this study, the experimental results above clearly show that the application of TOPSIS to the baseline methods results in better accuracy. Although the general accuracy of the proposed method is less than 0.5 in term of precision, the accuracy of all baseline methods is lower than that of the proposed method. For instance, the precision of the baseline methods does not exceed 0.1, except for NHSM, which scored around 0.12 using 100K MovieLens. In contrast, the maximum precision when TOPSIS was applied to NHSM reached 0.44 and 0.38 on the 100K and 1M MovieLens datasets, respectively. The low accuracy of the baseline methods in this case is related to the prediction algorithm. The prediction algorithm produces a predicted score for all candidate items within a given range of 1–5. Thus, there is a possibility that many items will have the same predicted score rating. Consequently, we do not know which (if either) of two items that have the same prediction score is actually more preferred by the user. This may lead to incorrect rankings and, in turn, low accuracy. However, the proposed method based on TOPSIS successfully minimizes the negative effect of the prediction algorithm in evaluating and ranking the candidate items. Thus, the application of TOPSIS significantly improves the accuracy of memory-based CF and produces more accurate results than the baseline methods.

## Conclusions

This paper has presented a new memory-based CF in which the TOPSIS method is applied to improve the accuracy of recommendations. The proposed method applies TOPSIS as a substitute for the prediction methods used in conventional memory-based CF. The application of TOPSIS to several commonly used CF methods (PCC, CPCC, SPCC, Cos, MSD, JMSD, and NHSM) was shown to produce sharp improvements in terms of precision, recall, F-measure, and MAP results over the respective baseline methods. In particular, the recall and MAP improved by a factor of more than two under application to the PCC, CPCC, SPCC, MSD, and Cos methods and by factors of more than three and four under application to JMSD and NHSM, respectively. Although the improvement in precision was generally smaller across all cases, applying TOPSIS achieved a three-fold increase in precision in NHSM and a doubling of the precision in the other methods. The results conclusively underline the enhancements that can be achieved by using TOPSIS in place of prediction to improve the accuracy of memory-based CF methods. This improvement arises from the consideration by the TOPSIS-enhanced CF of the item ratings by all *K*-neighbors in constructing a decision matrix to weight the criteria applied by the target user, and the application of the TOPSIS technique to evaluate and rank candidate items.

The key to successful memory-based CF is finding an appropriate set of neighbors. In future work, therefore, we will focus on improving the accuracy of recommendations by formulating a new similarity measure to locate sets of neighbors that produce better recommendations.

## Supporting information

S1 File Dataset. (ZIP)

## References

[1] Su X, Khoshgoftaar TM. A survey of collaborative filtering techniques. Advances in Artificial Intelligence. 2009;2009 10.1155/2009/421425

[2] Chen L, Chen GL, Wang F. Recommender systems based on user reviews: the state of the art. User Modeling and User-Adapted Interaction. 2015;25(2):99–154. 10.1007/s11257-015-9155-5

[3] Resnick P, Iacovou N, Suchak M, Bergstrom P, Riedl J, editors. GroupLens: an open architecture for collaborative filtering of netnews. Proceedings of the 1994 ACM conference on Computer Supported Cooperative Work; 1994 October 22–26, 1994 Chapel Hill, North Carolina, USA: ACM. 10.1145/192844.192905

[4] Hill W, Stead L, Rosenstein M, Furnas G, editors. Recommending and evaluating choices in a virtual community of use. Proceedings of the SIGCHI conference on Human Factors in Computing Systems; 1995: ACM Press/Addison-Wesley Publishing Co.

[5] Shardanand U, Maes P, editors. Social information filtering: algorithms for automating “word of mouth”. Proceedings of the SIGCHI conference on Human Factors in Computing Systems; 1995 May 07–11, 1995 Denver, Colorado, USA ACM Press/Addison-Wesley Publishing Co. 10.1145/223904.223931

[6] Balabanović M, Shoham Y. Fab: content-based, collaborative recommendation. Communications of the ACM. 1997;40(3):66–72.

[7] Konstan JA, Miller BN, Maltz D, Herlocker JL, Gordon LR, Riedl J. GroupLens: applying collaborative filtering to Usenet news. Communications of the ACM. 1997;40(3):77–87. 10.1145/245108.245126

[8] Resnick P, Varian HR. Recommender systems. Communications of the ACM. 1997;40(3):56–8. 10.1145/245108.245121

[9] Murti YR, Baizal Z. Compound Critiquing for Conversational Recommender System Based on Functional Requirement. Advanced Science Letters. 2016;22(8):1892–6.

[10] Haruna K, Ismail MA, Damiasih D, Sutopo J, Herawan T. A collaborative approach for research paper recommender system. PLoS ONE. 2017;12(10):e0184516 10.1371/journal.pone.0184516 28981512 PMC5628815

[11] Koohi H, Kiani K. A new method to find neighbor users that improves the performance of Collaborative Filtering. Expert Systems with Applications. 2017;83:30–9.

[12] Ricci F, Rokach L, Shapira B. Introduction to recommender systems handbook: Springer; 2011.

[13] Yang X, Guo Y, Liu Y, Steck H. A survey of collaborative filtering based social recommender systems. Computer Communications. 2014;41:1–10.

[14] Adomavicius G, Tuzhilin A. Toward the next generation of recommender systems: A survey of the state-of-the-art and possible extensions. IEEE Transactions on Knowledge and Data Engineering. 2005;17(6):734–49. 10.1109/Tkde.2005.99

[15] Sarwar B, Karypis G, Konstan J, Riedl J. Application of dimensionality reduction in recommender system-a case study. ACM WebKDD 2000 Workshop: DTIC Document; 2000. Report No.: TR 00–043.

[16] Chen L, Chen G, Wang F. Recommender systems based on user reviews: the state of the art. User Modeling and User-Adapted Interaction. 2015;25(2):99–154.

[17] Sharma L, Gera A. A survey of recommendation system: Research challenges. International Journal of Engineering Trends and Technology (IJETT). 2013;4(5):1989–92.

[18] Burke R. Hybrid web recommender systems The adaptive web: Springer; 2007 p. 377–408.

[19] Konstan JA, Riedl J, Borchers A, Herlocker JL, editors. Recommender systems: A GroupLens perspective. Recommender Systems: Papers from the 1998 Workshop (AAAI Technical Report WS-98-08); 1998.

[20] Bobadilla J, Ortega F, Hernando A, Gutiérrez A. Recommender systems survey. Knowledge-Based Systems. 2013;46:109–32.

[21] Lü L, Medo M, Yeung CH, Zhang Y-C, Zhang Z-K, Zhou T. Recommender systems. Physics Reports. 2012;519(1):1–49. 10.1016/j.physrep.2012.02.006

[22] Pazzani MJ, Billsus D. Content-based recommendation systems The adaptive web: Springer; 2007 p. 325–41.

[23] Mooney RJ, Roy L, editors. Content-based book recommending using learning for text categorization Proceedings of the fifth ACM conference on Digital Libraries; 2000 6 02–07, 2000 San Antonio, Texas, USA: ACM 10.1145/336597.336662

[24] Aggarwal CC. Content-Based Recommender Systems Recommender Systems: Springer; 2016 p. 139–66.

[25] Van den Oord A, Dieleman S, Schrauwen B, editors. Deep content-based music recommendation. Advances in Neural Information Processing Systems; 2013.

[26] Achakulvisut T, Acuna DE, Ruangrong T, Kording K. Science Concierge: A fast content-based recommendation system for scientific publications. PLoS ONE. 2016;11(7):e0158423 10.1371/journal.pone.0158423 27383424 PMC4934767

[27] Schafer JB, Frankowski D, Herlocker J, Sen S. Collaborative filtering recommender systems The adaptive web: Springer; 2007 p. 291–324.

[28] Ekstrand MD, Riedl JT, Konstan JA. Collaborative filtering recommender systems. Foundations and Trends in Human-Computer Interaction. 2011;4(2):81–173.

[29] Sarwar B, Karypis G, Konstan J, Riedl J, editors. Item-based collaborative filtering recommendation algorithms. Proceedings of the 10th international conference on World Wide Web; 2001 May 01–05, 2001 Hong Kong, Hong Kong: ACM. 10.1145/371920.372071

[30] Herlocker JL, Konstan JA, Terveen K, Riedl JT. Evaluating collaborative filtering recommender systems. ACM Transactions on Information Systems. 2004;22(1):5–53. 10.1145/963770.963772

[31] Kim H-N, Ji A-T, Ha I, Jo G-S. Collaborative filtering based on collaborative tagging for enhancing the quality of recommendation. Electronic Commerce Research and Applications. 2010;9(1):73–83.

[32] Iijima J, Ho S. Common structure and properties of filtering systems. Electronic Commerce Research and Applications. 2007;6(2):139–45.

[33] Zhang R, Liu Q-d, Wei J-X, editors. Collaborative Filtering for Recommender Systems. Advanced Cloud and Big Data (CBD), 2014 Second International Conference on; 2014: IEEE.

[34] Liao C-L, Lee S-J. A clustering based approach to improving the efficiency of collaborative filtering recommendation. Electronic Commerce Research and Applications. 2016;18:1–9.

[35] Cheng W, Yin G, Dong Y, Dong H, Zhang W. Collaborative filtering recommendation on users’ interest sequences. PLoS ONE. 2016;11(5):e0155739 10.1371/journal.pone.0155739 27195787 PMC4873175

[36] Adomavicius G, Tuzhilin A. Toward the next generation of recommender systems: A survey of the state-of-the-art and possible extensions. IEEE Transactions on Knowledge and Data Engineering. 2005;17(6):734–49.

[37] Herlocker JL, Konstan JA, Borchers A, Riedl J, editors. An algorithmic framework for performing collaborative filtering. Proceedings of the 22nd annual international ACM SIGIR conference on Research and Development in Information Retrieval; 1999 August 15–19, 1999; Berkeley, California, USA ACM. 10.1145/312624.312682

[38] Ahn HJ. A new similarity measure for collaborative filtering to alleviate the new user cold-starting problem. Information Sciences. 2008;178(1):37–51.

[39] Bobadilla J, Serradilla F, Bernal J. A new collaborative filtering metric that improves the behavior of recommender systems. Knowledge-Based Systems. 2010;23(6):520–8.

[40] Bobadilla J, Ortega F, Hernando A. A collaborative filtering similarity measure based on singularities. Information Process Management. 2012;48(2):204–17. 10.1016/j.ipm.2011.03.007

[41] Zang X, Liu T, Qiao S, Gao W, Wang J, Sun X, et al., editors. A New Weighted Similarity Method Based on Neighborhood User Contributions for Collaborative Filtering. Data Science in Cyberspace (DSC), IEEE International Conference on; 2016: IEEE.

[42] Bilge A, Yargıç A. Improving Accuracy of Multi-Criteria Collaborative Filtering by Normalizing User Ratings. Nadolu Üniversitesi Bilim Ve Teknoloji Dergisi A-Uygulamalı Bilimler ve Mühendislik. 2017;18(1):225–37.

[43] Liu H, Hu Z, Mian A, Tian H, Zhu X. A new user similarity model to improve the accuracy of collaborative filtering. Knowledge-Based Systems. 2014;56:156–66.

[44] Saranya KG, Sadhasivam GS. Modified Heuristic Similarity Measure for Personalization using Collaborative Filtering Technique. Applied Mathematics and Information Sciences. 2017;11(1):317–25. doi: 10.18576/amis/110137

[45] Patra BK, Launonen R, Ollikainen V, Nandi S. A new similarity measure using Bhattacharyya coefficient for collaborative filtering in sparse data. Knowledge-Based Systems. 2015;82:163–77.

[46] Jamali M, Ester M, editors. Trustwalker: a random walk model for combining trust-based and item-based recommendation Proceedings of the 15th ACM SIGKDD international conference on Knowledge Discovery and Data Mining; 2009 6 28–July 01, 2009; Paris, France: ACM 10.1145/1557019.1557067

[47] Zha Y, Zhai Y, editors. An Improved Collaborative Filtering Model Considering Item Similarity. Information Science and Cloud Computing Companion (ISCC-C), 2013 International Conference on; 2013: IEEE.

[48] Shen L, Zhou Y, editors. A new user similarity measure for collaborative filtering algorithm. Computer Modeling and Simulation, 2010 ICCMS'10 Second International Conference on; 2010: IEEE.

[49] Yang J, Kim J, Kim W, Kim YH. Measuring user similarity using electric circuit analysis: Application to collaborative filtering. PLoS ONE. 2012;7(11):e49126 10.1371/journal.pone.0049126 23145095 PMC3492312

[50] Gan M. Walking on a user similarity network towards personalized recommendations. PLoS ONE. 2014;9(12):e114662 10.1371/journal.pone.0114662 25489942 PMC4260921

[51] Al-Bashiri H, Abdulgabber MA, Romli A, Salehudin N. A Developed Collaborative Filtering Similarity Method to Improve the Accuracy of Recommendations under Data Sparsity. International Journal of Advanced Computer Science and Applications (IJACSA). 2018; 9(4):135–42. http://dx.doi.org/10.14569/IJACSA.2018.090423

[52] Cai G, Lv R, Wu H, Hu X, editors. An Improved Collaborative Method for Recommendation and Rating Prediction. Data Mining Workshop (ICDMW), 2014 IEEE International Conference on; 2014: IEEE.

[53] Zhang R, Liu Q-d, Gui C, Wei J-X, Ma H, editors. Collaborative filtering for recommender systems. Advanced Cloud and Big Data (CBD), 2014 Second International Conference on; 2014: IEEE.

[54] Iç YT, Yurdakul M. Development of a quick credibility scoring decision support system using fuzzy TOPSIS. Expert Systems with Applications. 2010;37(1):567–74.

[55] Goldberg D, Nichols D, Oki BM, Terry D. Using collaborative filtering to weave an information tapestry. Communications of the ACM. 1992;35(12):61–70.

[56] Polatidis N, Georgiadis CK. A multi-level collaborative filtering method that improves recommendations. Expert Systems with Applications. 2016;48:100–10.

[57] Huang B-H, Dai B-R, editors. A Weighted Distance Similarity Model to Improve the Accuracy of Collaborative Recommender System. Mobile Data Management (MDM), 2015 16th IEEE International Conference on; 2015 15–18 June 2015; Pittsburgh, PA, USA: IEEE. 10.1109/MDM.2015.43

[58] Breese JS, Heckerman D, Kadie C, editors. Empirical analysis of predictive algorithms for collaborative filtering. Proceedings of the Fourteenth conference on Uncertainty in Artificial Intelligence; 1998: Morgan Kaufmann Publishers Inc.

[59] Ungar LH, Foster DP, editors. Clustering methods for collaborative filtering. AAAI Workshop on Recommendation Systems; 1998.

[60] Hofmann T. Latent semantic models for collaborative filtering. ACM Transactions on Information Systems (TOIS). 2004;22(1):89–115. 10.1145/963770.963774

[61] Koren Y, Bell R, Volinsky C. Matrix factorization techniques for recommender systems. Computer. 2009(8):30–7.

[62] Zeng W, Zeng A, Liu H, Shang M-S, Zhang Y-C. Similarity from multi-dimensional scaling: Solving the accuracy and diversity dilemma in information filtering. PLoS ONE. 2014;9(10):e111005 10.1371/journal.pone.0111005 25343243 PMC4208813

[63] Al-bashiri H, Abdulgabber MA, Romli A, Hujainah F, editors. Collaborative Filtering Similarity Measures: Revisiting. Proceedings of the International Conference on Advances in Image Processing; 2017: ACM. 10.1166/asl.2017.10020

[64] Al-Bashiri H, Abdulgabber MA, Romli A, Hujainah F. Collaborative Filtering Recommender System: Overview and Challenges. Advanced Science Letters. 2017;23(9):9045–9. 10.1166/asl.2017.10020

[65] Mahara T. A New Similarity Measure Based on Mean Measure of Divergence for Collaborative Filtering in Sparse Environment. Procedia Computer Science. 2016;89:450–6.

[66] Liu HF, Hu Z, Mian A, Tian H, Zhu XZ. A new user similarity model to improve the accuracy of collaborative filtering. Knowledge-Based Systems. 2014;56:156–66. 10.1016/j.knosys.2013.11.006

[67] Koutrika G, Bercovitz B, Garcia-Molina H, editors. FlexRecs: expressing and combining flexible recommendations. Proceedings of the 2009 ACM SIGMOD International Conference on Management of Data; 2009 June 29–July 02, 2009 Providence, Rhode Island, USA ACM. 10.1145/1559845.1559923

[68] Liu J-H, Zhou T, Zhang Z-K, Yang Z, Liu C, Li W-M. Promoting cold-start items in recommender systems. PLoS ONE. 2014;9(12):e113457 10.1371/journal.pone.0113457 25479013 PMC4257537

[69] Bobadilla J, Hernando A, Ortega F, Bernal J. A framework for collaborative filtering recommender systems. Expert Systems with Applications. 2011;38(12):14609–23. 10.1016/j.eswa.2011.05.021

[70] Kahraman C. Multi-criteria decision making methods and fuzzy sets Fuzzy Multi-Criteria Decision Making: Springer; 2008 p. 1–18.

[71] Xu L, Yang J-B. Introduction to multi-criteria decision making and the evidential reasoning approach: Manchester School of Management; 2001.

[72] Hwang C-L, Yoon K. Multiple attribute decision making: methods and applications a state-of-the-art survey: Springer Science & Business Media; 2012.

[73] Yoon K, Hwang C. TOPSIS (technique for order preference by similarity to ideal solution)–a multiple attribute decision making, w: Multiple attribute decision making–methods and applications, a state-of-the-art survey Berlin: Springer Verlag; 1981.

[74] Shih H-S, Shyur H-J, Lee ES. An extension of TOPSIS for group decision making. Mathematical and Computer Modelling. 2007;45(7):801–13.

[75] Parkan C, Wu M. On the equivalence of operational performance measurement and multiple attribute decision making. International Journal of Production Research. 1997;35(11):2963–88.

[76] Olson DL. Comparison of weights in TOPSIS models. Mathematical and Computer Modelling. 2004;40(7–8):721–7.

[77] Ishizaka A, Nemery P. Multi-criteria decision analysis: methods and software: John Wiley & Sons; 2013.

[78] MovieLens | GroupLens 2013 [updated 2013-09-06]. Available from: https://grouplens.org/datasets/movielens/.

[79] Cantador I, Brusilovsky PL, Kuflik T. Second workshop on information heterogeneity and fusion in recommender systems (HetRec2011): ACM; 2011.

[80] Guo G, Zhang J, Yorke-Smith N, editors. A Novel Bayesian Similarity Measure for Recommender Systems. International Joint Conference on Artificial Intelligence; 2013.

[81] Han J, Pei J, Kamber M. Data mining: concepts and techniques: Elsevier; 2011.

[82] Sarwar B, Karypis G, Konstan J, Riedl J, editors. Analysis of recommendation algorithms for e-commerce. Proceedings of the 2nd ACM conference on Electronic Commerce; 2000: ACM.

[83] Choi K, Suh Y. A new similarity function for selecting neighbors for each target item in collaborative filtering. Knowledge-Based Systems. 2013;37:146–53.

[84] Basu C, Hirsh H, Cohen W, editors. Recommendation as classification: Using social and content-based information in recommendation AAAI/IAAI; 1998.

[85] Salton G, McGill MJ. Introduction to modern information retrieval McGraw-Hill New York; 1986.

[86] Baeza-Yates R, Ribeiro-Neto B. Modern information retrieval: ACM Press New York; 1999.

